# Phylodynamic Analysis of the Emergence and Epidemiological Impact of Transmissible Defective Dengue Viruses

**DOI:** 10.1371/journal.ppat.1003193

**Published:** 2013-02-28

**Authors:** Ruian Ke, John Aaskov, Edward C. Holmes, James O. Lloyd-Smith

**Affiliations:** 1 Department of Ecology and Evolutionary Biology, University of California, Los Angeles, California, United States of America; 2 Institute of Health and Biomedical Innovation, Queensland University of Technology, Brisbane, Australia; 3 School of Biological Sciences and Sydney Medical School, The University of Sydney, Sydney, Australia; 4 Fogarty International Center, National Institutes of Health, Bethesda, Maryland, United States of America; University of Texas at Austin, United States of America

## Abstract

Intra-host sequence data from RNA viruses have revealed the ubiquity of defective viruses in natural viral populations, sometimes at surprisingly high frequency. Although defective viruses have long been known to laboratory virologists, their relevance in clinical and epidemiological settings has not been established. The discovery of long-term transmission of a defective lineage of dengue virus type 1 (DENV-1) in Myanmar, first seen in 2001, raised important questions about the emergence of transmissible defective viruses and their role in viral epidemiology. By combining phylogenetic analyses and dynamical modeling, we investigate how evolutionary and ecological processes at the intra-host and inter-host scales shaped the emergence and spread of the defective DENV-1 lineage. We show that this lineage of defective viruses emerged between June 1998 and February 2001, and that the defective virus was transmitted primarily through co-transmission with the functional virus to uninfected individuals. We provide evidence that, surprisingly, this co-transmission route has a higher transmission potential than transmission of functional dengue viruses alone. Consequently, we predict that the defective lineage should increase overall incidence of dengue infection, which could account for the historically high dengue incidence reported in Myanmar in 2001–2002. Our results show the unappreciated potential for defective viruses to impact the epidemiology of human pathogens, possibly by modifying the virulence-transmissibility trade-off, or to emerge as circulating infections in their own right. They also demonstrate that interactions between viral variants, such as complementation, can open new pathways to viral emergence.

## Author Summary

Defective viruses are viral particles with genetic mutations or deletions that eliminate essential functions, so that they cannot complete their life cycles independently. They can reproduce only by co-infecting host cells with functional viruses and ‘borrowing’ their functional elements. Defective viruses have been observed for many human pathogens, but they have not been thought to impact epidemiological outcomes. Recently it was reported that a lineage of defective dengue virus spread through humans and mosquitoes in Myanmar for at least 18 months in 2001–2002. In this study, we investigate the emergence and epidemiological impact of this defective lineage by combining genetic sequence analyses with mathematical models. We show that the defective lineage emerged from circulating dengue viruses between June 1998 and February 2001, and that it spreads because—surprisingly—its presence causes functional dengue viruses to transmit more efficiently. Our model shows that this would cause a substantial rise in total dengue infections, consistent with historically high levels of dengue cases reported in Myanmar during 2001 and 2002. Our study yields new insights into the biology of dengue virus, and demonstrates a previously unappreciated potential for defective viruses to impact the epidemiology of infectious diseases.

## Introduction

Although the high deleterious mutation rate of RNA viruses ensures that many genomes are defective [1], the long-term evolutionary and epidemiological consequences of the presence and transmission of defective viruses rarely have been discussed. To date, most work has focused on laboratory studies of defective-interfering (DI) viruses (or particles); these are characterized by major deletion mutations which give them a replication advantage over full-length viral genomes [2]–[3]. DI particles interfere with the functional virus by competition for materials essential for replication and transmission, such as polymerase enzymes or capsid proteins [4]. It has been hypothesized that, by limiting the production of the functional virus, DI particles may play an important role in persistent infections [5] and could even serve as therapies for viral infections [6]–[8]. Recently, defective viruses with full-length genomes also have been recognized as a general phenomenon in many major human pathogens, including human immunodeficiency virus, hepatitis B virus, hepatitis C virus, West Nile virus, and dengue virus [2], [9]–[16]. However, the role they play in natural viral populations is unclear [17] and an epidemiological impact has not been demonstrated.

It was shown recently that a defective dengue virus (DENV) lineage, characterized by a full-length genome and truncated E protein, was able to able to pass between individuals in a natural transmission cycle involving humans and mosquitoes during 2001 and 2002 in Myanmar [14]. Dengue virus is a vector-borne RNA virus that is transmitted between humans and mosquitoes, and infects 50–100 million people globally each year. There are four serotypes of dengue virus (DENV-1 to 4), all of which are endemic in Myanmar [18]. This lineage of defective viruses arose from a point mutation that introduced a stop codon in the viral envelope (E) glycoprotein gene of DENV-1. It transmitted persistently for at least 18 months, increased markedly in frequency from 2001 to 2002, and also spread thousands of kilometers to other geographic areas such as New Caledonia and Singapore [14]. Although defective viruses are frequently found in dengue patients [3], [14], [19], their long-term transmission was surprising since they were not thought to transmit naturally between hosts.

The most likely explanation for the persistence of the defective DENV genomes in nature is complementation with a fully competent strain of the same virus (here termed the ‘functional virus’) in dually infected cells. The strict requirement for complementation by the functional virus means that sustained transmission of defective particles requires frequent infection of host individuals (and host cells) by both types of particles. Given that the phenotypic effect most commonly associated with defective particles is reduced production of the functional virus due to interference, the mechanisms responsible for the emergence and spread of this defective lineage of DENV-1 are mysterious. Intriguingly, Myanmar saw historically high levels of reported dengue cases during the period when the transmitted defective particle (tDP) was reported [18].

In this study, we address the questions of how the tDP emerged and spread and evaluate its relationship with dengue transmission using a phylodynamic approach [20]–[21]. This approach unifies phylogenetic and dynamic modeling techniques to analyze genetic and epidemiological data, and thus is an important tool for the study of emerging viruses. The emergence of the tDP lineage also highlights several general research themes regarding viral emergence. Successful sustained transmission of a newly emerged viral strain depends on a complicated interplay between evolutionary and ecological processes [22]. On one hand, the high mutation rate and short generation time of RNA viruses mean that evolutionary processes occur rapidly, and evolutionary outcomes depend on the fitness of viral mutants at different stages of viral life-cycles and, sometimes, on the interactions between viral variants [23]–[27]. On the other hand, ecological and epidemiological factors, such as host contact patterns [28]–[29], transmission routes [30] and host movements [31]–[32], also determine the success of viral transmission in a population. The unexpected finding of dengue tDPs raises questions about how these factors interacted to shape the emergence of the tDP, and what impact the defective lineage may have had on the epidemiology of competent dengue strains. These questions highlight gaps in our understanding of the possible roles played by defective particles in the transmission biology of all viruses, and the potential for defective particles to emerge as circulating infections in their own right, i.e. always super-infecting over a functional virus, in the manner of satellite viruses [17]. More broadly, the example of tDPs offers opportunities to examine the roles that factors such as complementation and interaction between viral variants play in the process of viral emergence.

## Results

### The transmitted defective DENV particle emerged between June 1998 and February 2001

To examine the evolution of the tDP lineage with its associated (functional) DENV-1 lineages, we first categorized the sequences reported by Aaskov *et al*. [14] into the three distinct lineages identified in their study. We denote the three lineages as the stop-codon lineage (the lineage consisting of the tDP isolates), the wt-1 lineage (the functional DENV-1 lineage associated with the stop-codon lineage) and the wt-2 lineage. Within each host, most values of the ratio of nonsynonymous to synonymous substitutions per site (d_N_/d_S_) for E genes from the stop-codon lineage are close to 1, indicating neutral evolution of the defective lineage; in contrast, all the d_N_/d_S_ values calculated from the competent lineages are below 1, indicating purifying selection acting on the competent lineages (Table S1). This is consistent with typical characteristics of defective particles and the conclusion that the E gene of tDPs does not encode functional protein [14]. We further examined the sequences of 24 isolates from a patient in New Caledonia sampled in February 2003 [13], and found that, among the 24 sequences, seven belong to the stop-codon lineage and four belong to the wt-1 lineage, confirming that the defective isolates and some functional isolates were derived from the lineages circulating in Myanmar.

**Figure 1:**
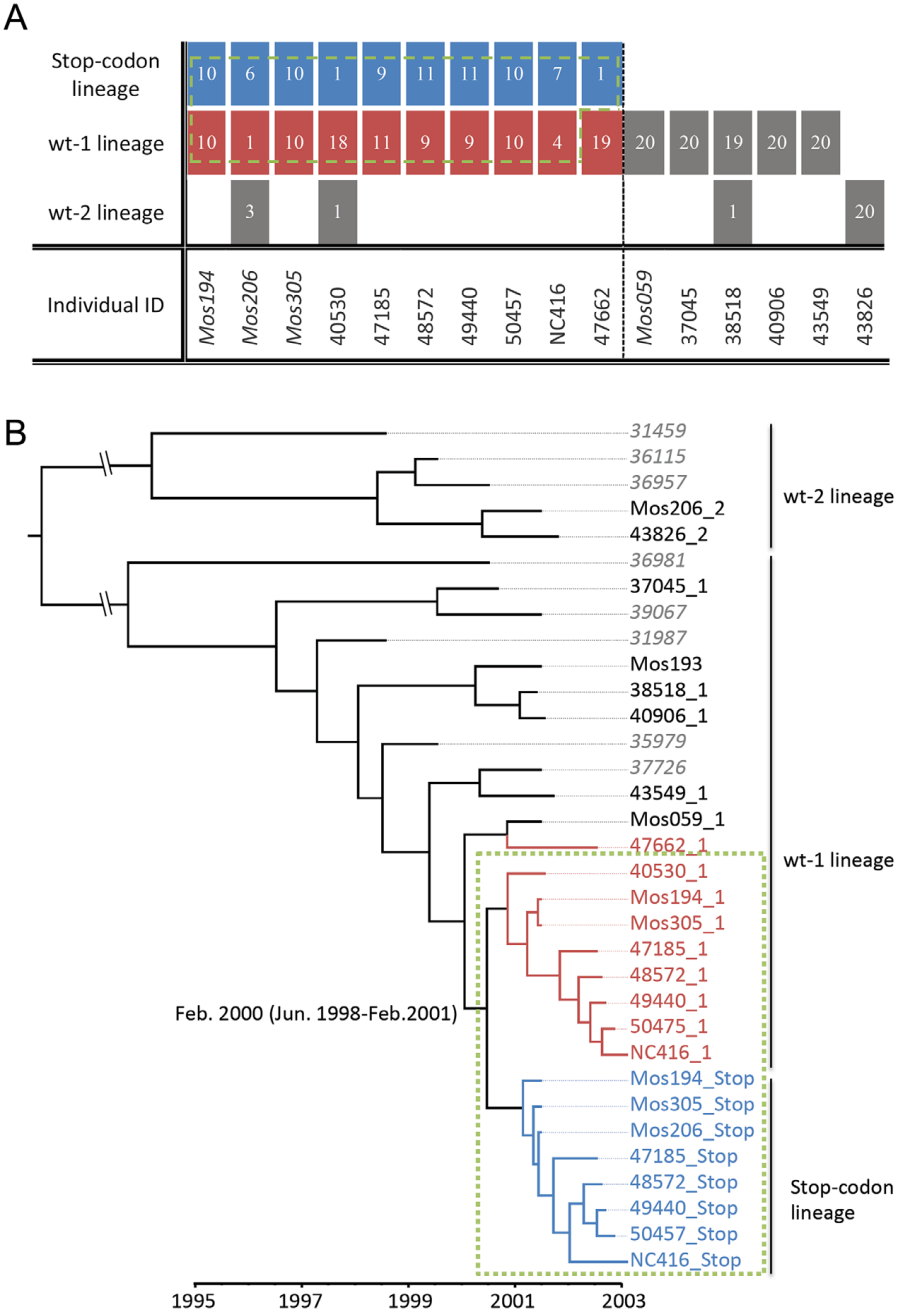
The transmitted defective particles (tDP) are detected in individuals infected by functional viruses that are genetically close related to the stop-codon lineage. (**A**) Table showing the number of sequences belonging to each lineage, from the multiple isolates derived from each individual. (**B**) The evolutionary and transmission dynamics of DENV-1 isolates revealed by the MCC tree. The scale bar shows the time in years when the samples were taken. The numbers at the root of the stop-codon clade of the tree show the estimated time in years (and 95% credible intervals) of the time to common ancestry of the stop-codon lineage and the wt-1 lineage. The stop-codon lineage sequences are color-coded in blue, and the wt-1 lineage sequences that were isolated from tDP infected individuals are in red. The green dashed box (in both panels) shows the sequences that are descendants of the functional DENV-1 genotype that generated the stop-codon lineage. The patient numbers of the consensus sequences derived from multiple intra-host viral isolates are followed by their lineage identification, i.e. ‘_1’,‘_2’ and ‘_stop’. The patient numbers of the 8 additional sequences are in gray italic font.

To estimate the time of tDP emergence, we first derived consensus sequences for the wt-1 viral lineage and the stop-codon lineage in each individual where more than one viral isolate was available (Fig. 1A). To cover a broader time period in our estimation, we randomly selected eight additional DENV-1 sequences isolated in Myanmar during 1998 and 2001 [18]. A Maximum Clade Credibility (MCC) tree estimated using BEAST [33] showed that, as expected, all stop-codon sequences fall in the same clade of the tree, and that both the functional viruses and the tDP detected in New Caledonia were transmitted from Myanmar (Fig. 1B). The estimated time of divergence of the stop-codon lineage from the wt-1 lineage is February 2000 (95% Highest Probability Density: June 1998 to February 2001).

The MCC tree (Fig. 1B) revealed two additional interesting features. First, the consensus sequences of wt-1 viruses isolated from tDP-infected individuals are closely related to the stop-codon lineage (red branches in Fig. 1B). In fact, all those sequences except individual 47662 (47662_1, where ‘1’ denotes the wt-1 lineage) form a monophyletic group with sequences of the stop-codon lineage (green box in Fig. 1B), suggesting they are derived from a common ancestor. (A tree topology test showed that alternative trees in which sequence 47662_1 belongs to this monophyletic group cannot be excluded statistically; see Fig. S1 and Table S2). This clustering pattern suggests that the DENV-1 lineage isolated from dually infected individuals shares the same transmission history as the stop-codon lineage. This would be expected if the tDP co-transmits with functional DENV-1, i.e. if both DENV-1 viruses and tDPs are transmitted simultaneously to new hosts in the same contact event. The second interesting feature is that the stop-codon lineage rose from being quite rare in 2001 to being found in all sampled patients infected by DENV-1 in 2002 (Fig. 1B), indicating a possible transmission advantage for this stop-codon lineage. Below, using dynamical models, we provide evidence that tDP is primarily co-transmitted with DENV-1 and that this co-transmission increases transmission fitness, thereby allowing tDP to rise to a high frequency in the population.

### tDP spread is driven by efficient co-transmission to susceptible individuals

**Figure 2:**
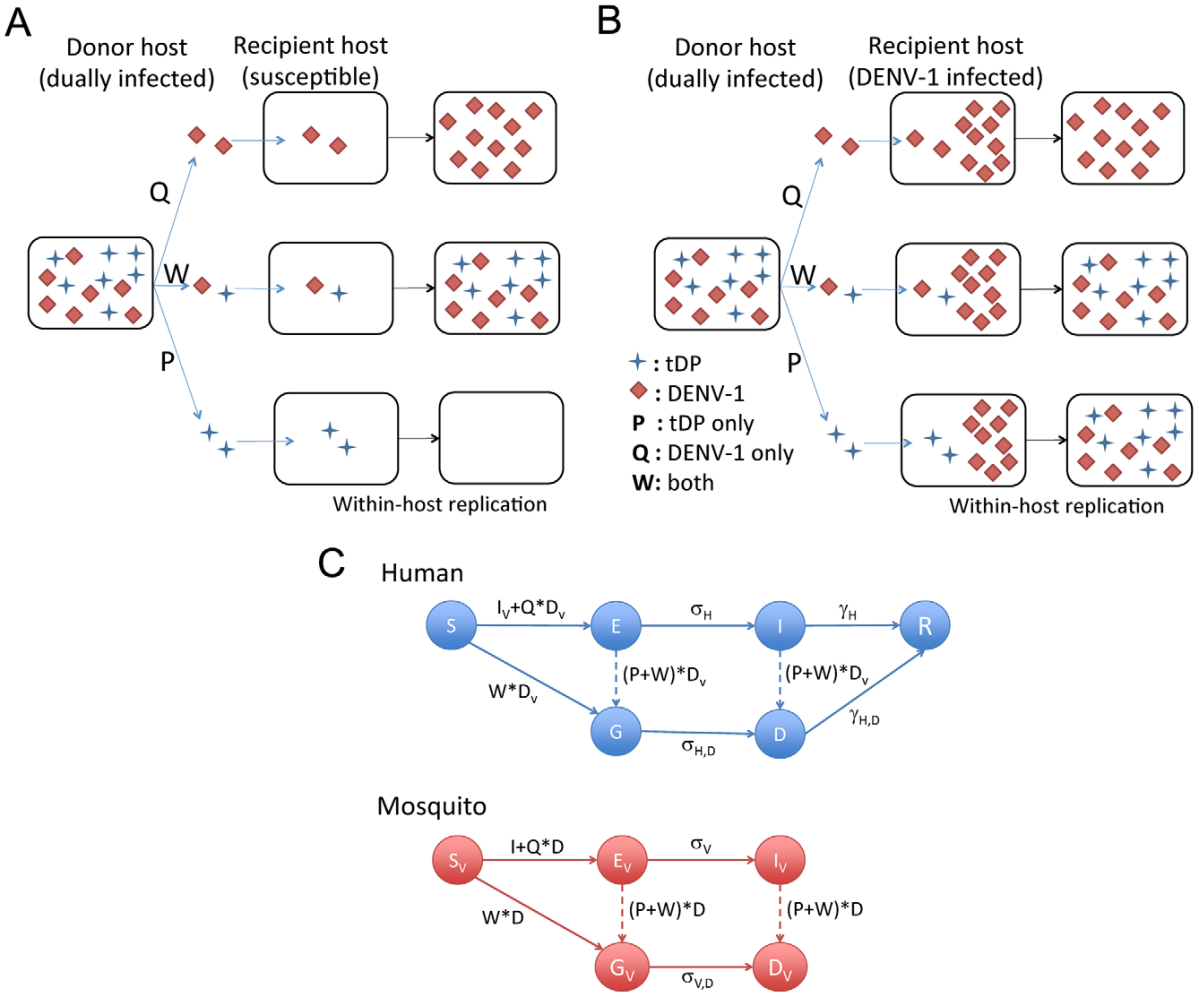
Schematic diagrams for different transmission routes of tDP and the compartmental model for tDP and DENV-1 transmission. (**A and B**) Two possible routes of tDP transmission. The tDP can replicate only in dually infected individuals, so these individuals are the only source for transmission of tDP. We consider situations where a dually infected ‘donor host’ contacts a ‘recipient host’ that is either uninfected and completely susceptible (in panel A) or infected with the functional virus (DENV-1) only (in panel B). By chance, the viral population that is transmitted to the recipient host may contain the tDPs only (P), the functional virus only (Q), or both types of particles (W). If the transmitted viral population consists of only the functional virus, the recipient host would become a DENV-1-only infected individual. If this viral population consists of both types of particles, it is possible for the recipient host to become a dually infected individual, irrespective of whether the recipient host is already infected or not. However, if the transmitted viral population consists of only tDP particles, the recipient host can become dually infected only if it is already infected with DENV-1 as shown in panel B (a pathway we call super-infection). (**C**) Schematic for the compartmental model of DENV-1 and tDP transmission dynamics. S, E, I and R denote the susceptible, exposed, infectious, recovered compartments for the functional virus, respectively. G and D are the exposed and infectious compartments for dual infection, respectively. *σ* and *γ* denote the rates at which latent individuals become infectious and infectious individuals recover, respectively. Dashed lines denote processes with rates that are orders of magnitude lower than rates shown with solid lines. The mathematical expressions show the infectious population groups that contribute to each route of transmission, and associated scaling parameters. Model equations are shown in Eqn. (1) of the Methods.

To identify potential mechanisms that allow for sustained transmission of the tDP, we constructed a seasonally forced dynamical model for the transmission dynamics of the tDP and DENV-1 by combining aspects of established models for dengue [34] and defective particles [7] (see Methods and Text S1). We first focus on the possible mechanisms of transmission of tDPs. The donor host (either human or mosquito) must be dually infected with DENV-1 and tDP, and we consider two types of contacts that may lead to tDP transmission (Fig. 2A and B): contact either with uninfected susceptible individuals (possibly leading to infection of the susceptible with one or both viruses) or with individuals infected with DENV-1 only (possibly leading to super-infection with tDP). Three types of transmission events are possible: transmission of tDP only (which matters only if the host is already infected with DENV-1), transmission of DENV-1 only, or transmission of both tDP and DENV-1. The rates at which these three alternatives occur, relative to the rate of transmission of the functional virus from DENV-1-infected hosts, are modeled using dimensionless scaling parameters *P*, *Q* and *W*, respectively (Fig. 2C). These three parameters incorporate the changes in viral transmission rates from dually infected human and mosquito individuals as a result of all relevant factors including changes in viral titers and host movement or behavior patterns.

**Figure 3:**
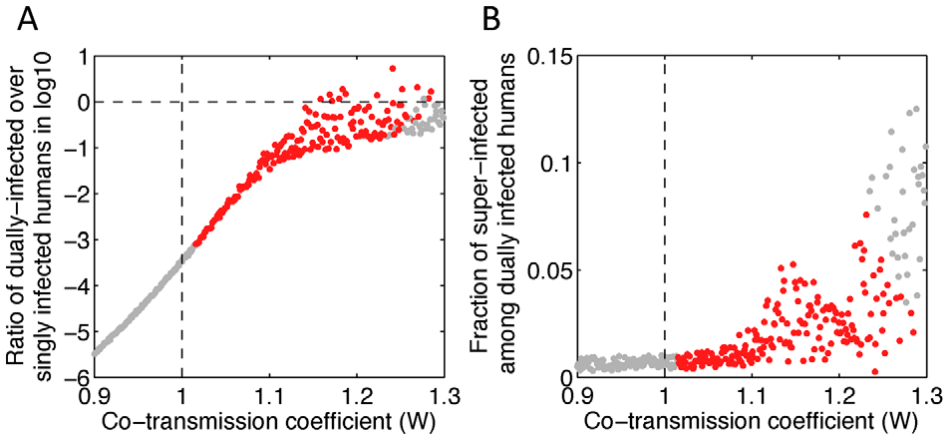
The tDP is primarily transmitted through the co-infection of susceptible individuals. (**A**) The ratio of the cumulative number of dually infected humans (D) over the cumulative number of humans infected with DENV-1 only (I) during the 2^nd^ and 3^rd^ years after tDP emergence (corresponding to calendar year 2001 and 2002) is plotted against the parameter *W* describing the efficiency of co-transmission. (**B**) The fraction of super-infected humans among all dually infected humans for different values of the co-transmission coefficient. Simulation results where tDP is and is not transmitted persistently during the 3^rd^ year of the simulation are denoted as red and gray dots, respectively. Vertical dashed lines denote the threshold of *W* = 1, whereas the horizontal dashed line in panel A denotes the threshold where the number of dually infected cases is equal to the number of singly infected cases. 1,000 samples of Latin hypercube sampling were performed with parameters *P* and *Q* sampled uniformly between 0 and 1 and *W* sampled uniformly between 0 and 1.3.

To investigate the key mechanisms contributing to tDP emergence and transmission, we simulated the model with different values of *P*, *Q* and *W* while holding other parameters constant. Note that the values of *P* and *Q*, i.e. transmission of tDP only and DENV-1 only, are probably small because of the high number of viruses thought to be transmitted between human and mosquito [35]. Nonetheless, we allow them to vary in a wide range (0 to 1) to be comprehensive. We found that the essential determinant of long-term transmission of the tDP is the value of parameter *W*, i.e. the efficiency of co-transmission of both the tDP and functional DENV-1. Continuous transmission of the tDP over multiple years requires that co-transmission of tDP and DENV-1 is more efficient than transmission of wild-type DENV-1 in the absence of tDP (i.e. *W*>1.0, irrespective of the values of *P* and *Q*, as shown in Fig. 3A). For the abundance of dually infected individuals (D) to rise to a level comparable to DENV-1 infected individuals (I) within 3 years, as observed in the data from Myanmar, the co-transmission of tDP and DENV-1 must be 15% more efficient than the wild-type transmission (*W*>1.15, red dots above the horizontal dashed line in Fig. 3A), averaging over humans and mosquitoes. An alternative explanation of the observed rise in frequency of dually infected individuals is genetic drift without any transmission advantage. To test the validity of our deterministic modeling approach, we conducted a stochastic analysis based on a Wright-Fisher model and found that the probability that the observed rise in frequency occurred due to purely neutral evolution is extremely small (see Text S1 for details). Therefore, from epidemiological arguments, transmission of tDP is driven primarily by co-transmission of tDP and DENV-1, which is more efficient than transmission of DENV-1 by singly-infected hosts. However, in simulations with considerably higher values of *W*, both DENV-1 and the tDP go extinct due to depletion of susceptible individuals during the post-epidemic refractory period.

To better understand the transmission biology of the defective virus, we evaluated the relative importance of the two mechanisms of transmission in driving tDP spread. By comparing the numbers of dually infected human individuals arising from each type of contact, we found that super-infection accounts for <8% of dually-infected cases (Fig. 3B), indicating that co-transmission of the tDP and DENV-1 to uninfected individuals is by far the dominant transmission route (Fig. 3B). The reason is that the number of DENV-1 infected individuals is much smaller than the number of susceptible individuals for both humans and mosquitoes in dengue-endemic areas [34], [36], and therefore the rate of contact between dually infected and DENV-1 infected individuals is too low to maintain sustained transmission by super-infection. Furthermore, the fraction of super-infected individuals in the simulation (Fig. 3B) is likely an overestimate, since other factors not considered in the model, such as super-infection exclusion, a process whereby an infected cell cannot be infected with the same or a closely related virus [37], may further restrict the frequency of super-infection events.

### Overall dengue transmission is increased in the presence of the tDP

To characterize the epidemiological conditions that allow the tDP to emerge and rise to high frequency in the infected population, we calculated the effective reproduction number, R_eff,co_ (see Eqn.2 in Methods), for the co-transmission route in a simplified model that ignores seasonal forcing and super-infection, i.e. transmission route denoted by dashed lines in Fig. 2C. We found that successful invasion of tDP (R_eff,co_>1) depends on the values of four parameters that characterize the dually infected individuals: the infectious period of dually infected humans (1/*γ_H,D_*), the incubation period of dually infected mosquitoes (1/*σ_V,D_*), and the relative efficiencies of co-transmission by dually infected humans (*W_H_*) and mosquitoes (*W_V_*). The overall co-transmission parameter *W*, analyzed in Fig. 3, is the geometric mean of *W_H_* and *W_V_*. Apart from parameters *W_H_* and *W_V_*, the dependence of R_eff,co_ on the parameters *γ_H,D_* and *σ_V,D_* arises from the altered durations of the infectious periods of dually infected humans and mosquitoes, respectively. Note that since dengue infections of mosquitoes are life-long, a shorter incubation period increases the time spent in the infectious state and therefore increases transmission potential.

**Figure 4:**
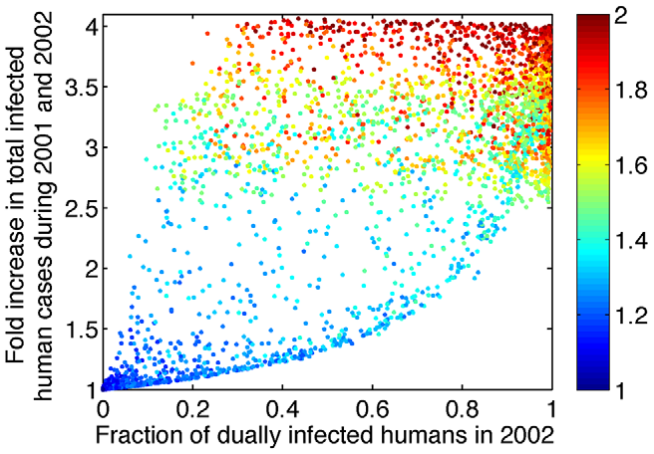
Increasing R_eff,co_ above 1 leads to increases in the fraction of dually infected individuals among all infected individuals and in the total number of infected individuals. The y-axis shows the predicted fold increase in total DENV-1 infected humans during 2001 and 2002 relative to the number of human cases in the absence of tDP. The x-axis shows the predicted fraction of human cases who were dually infected during 2002. The color of the dots indicates the value of R_eff,co_ as shown in the color bar. Only simulations with R_eff,co_>1 are shown; when R_eff,co_<1, the fraction of dually infected humans is near 0 and the fold increase in total dengue cases is near 1. 10,000 sets of parameter values were sampled using Latin hypercube sampling. *P* and *Q* were sampled from a uniform distribution between 0 and 1 and W was sampled from a uniform distribution between 0 and 2. *γ_H,D_* and *σ_V,D_* were sampled in a way such that the fold changes in human infectious period (*γ_H,D_/γ_H_*) and mosquito incubation period (*σ_V,D_/σ_V_*) range from 0.5 to 2. The values of *γ_H_*, *σ_V_* and *μ_V_* are kept constant.

To assess the possible epidemiological impact of tDP emergence, we simulated the full model from the emergence of tDP (assumed here to occur in year 2000) through the period for which we have data (to the end of 2002), for a range of biologically plausible parameter values (see Methods). We randomly sampled the four parameters that determine the value of R_eff,co_, and the two additional transmission parameters *P* and *Q*, and computed the value of R_eff,co_ for each simulation. When R_eff,co_<1, the fraction of human cases that were dually infected in year 2002 was negligible, and the total number of dengue cases during the three years after tDP emergence did not change appreciably from the number in the absence of tDP. In contrast, values of R_eff,co_>1 led to increases in both the fraction of dengue-infected humans who were dually infected and in the total number of dengue cases (Fig. 4). Interestingly, the model reveals a lower bound of the fold increase in total dengue cases for a given observed fraction of dually infected individuals. This is because increases in the fraction of dually infected individuals result from more efficient co-transmission, which also increases the total number of infected individuals. Aaskov *et al.* reported that 5 out of 5 human patients sampled in 2002 were dually infected [14]. With reference to the results in Fig. 4, the observation that all cases were dually infected in 2002 predicts a 2.5–4 fold increase in total dengue cases during 2001 and 2002, though of course the sample size is small so uncertainties are large. (For 5/5, the lower bound of the 95% C.I. for the proportion is 0.48, which corresponds to a lower bound of a 1.3 fold increase in total cases.)

To derive more robust estimates of the possible impact of tDP transmission on overall dengue transmission, we used a likelihood framework to estimate the value of R_eff,co_ based on previously reported data [14] and on our finding that tDP emerged between June 1998 and February 2001 (see Text S1). Since R_eff,co_ is influenced by the four parameters describing dual infections, as shown above, and the realized changes in these four parameters are unknown, we explored four scenarios where the changes in R_eff,co_ arise entirely from changes in each parameter. Maximum likelihood (ML) estimation was used to infer parameter values for each scenario, yielding an estimate of R_eff,co_, and the time of tDP emergence (*t_emg_*) was estimated simultaneously (see Fig. S2 for a comparison between data and simulation using ML parameter values). The same qualitative picture emerges for all four scenarios (Table 1): the ML estimates of R_eff,co_ fall in the range 1.24–1.28 (95% C.I.: 1.13–1.89), giving rise to a 2.3–3.2 (95% C.I.: 1.4–4.1) fold increase in overall DENV-1 cases during 2001 and 2002 as a result of tDP transmission. Sensitivity analysis (see ) showed that our results are robust to the assumed magnitude of seasonality in mosquito populations (Table S3 and S4) and phase of multi-annual cycles in dengue incidence (Table S5 and Fig. S3). For all scenarios analyzed, the ML estimate of R_eff,co_ falls between 1.2 and 1.3. Because of limited data and inherent challenges in fitting the non-stationary dynamics of a complex system, we interpret these results not as precise estimates but as confirmation of our qualitative conclusion that co-transmission of tDP and DENV-1 has a substantially increased transmission potential, which in turn is expected to lead to elevated incidence compared with DENV-1 alone.

**Table 1:** Maximum-likelihood estimates of parameter values and their corresponding increase in overall DENV-1 cases during 2001 and 2002.

Parameter	Value (C.I.*)	R_eff,co_	t_emg_	Neg. Log-Likelihood	Fold increase in DENV-1 cases during 2001–2002 (range of variation**)
**W_H_**	1.24 (1.13–1.89)	1.24 (1.13–1.89)	Dec, 1998	4.99	2.3 (1.4–3.6)
**W_V_**	1.24 (1.13–1.71)	1.24 (1.13–1.71)	Dec, 1998	5.26	2.4 (1.4–3.5)
**γ_H,D_**	0.131 day^−1^ (0.10–0.15)	1.28 (1.13–1.59)	Dec, 1998	5.81	3.2 (1.5–4.1)
**σ_V,D_**	0.186 day^−1^ (0.14–0.79)	1.24 (1.14–1.57)	Oct, 1998	4.88	2.5 (1.5–3.4)

* 95% confidence intervals that are calculated using parameter profiling.

** The ranges of variation are calculated using the estimated parameter values at the boundaries of the 95% confidence intervals.

## Discussion

Our results reveal a significant impact of transmissible defective particles (tDPs) on the epidemiological dynamics of dengue virus, a phenomenon that has not been reported previously for any human pathogen. We first showed that co-transmission of tDP and the functional virus to uninfected hosts is the primary mechanism of tDP transmission, and, unexpectedly, this co-transmission route has a higher transmission potential than the transmission of the functional virus only. This qualitative conclusion is robust to assumptions about parameter values and underlying epidemiology of dengue virus. Based on this higher transmission potential, our model predicts a substantial increase in the total DENV-1 incidence during 2001 and 2002, which is consistent with the historically large outbreaks reported in Myanmar during this period [18].

The finding that co-infection of previously uninfected individuals constitutes the primary transmission route sheds light on the biology of dengue infection and transmission. Successful establishment of defective particles in a newly infected host requires that dual infections of host cells occur frequently throughout the full course of infection, including in the initial stage. This indicates that a large number of virions must be transmitted, consistent with the idea of a relatively wide transmission bottleneck for dengue [9], [13]–[14]. It also suggests that the process of viruses entering host cells near the site of infection is highly constrained spatially, such that the infecting dose of virions is restricted to a relatively small number of host cells available for infection. To maintain the transmission chain, these conditions must hold for co-infections in both humans and mosquitoes, although the relevant mechanisms of infection are completely different.

Several different mechanisms could account for the higher transmission potential of dually infected hosts relative to singly infected hosts. It is possible that the higher transmission arises from intrinsic properties of the functional DENV-1 genotypes in dually infected individuals, and the defective lineage has no effect. In this case the unprecedented finding of a co-transmitted defective lineage (and 100% frequency of dual infection in 2002) is strictly coincidental, and has no causal relationship with the increased fitness of its associated DENV-1 lineage. We think this is unlikely. A more parsimonious explanation is that the tDP increases the transmission potential by modulating the within-host replication of DENV-1 from a non-optimal level. Previous work on the theory of virulence evolution suggests that there exists an optimal viral load that maximizes transmission potential [38]–[39]. Transmission increases with viral load when viral loads are low, but once viral loads exceed the optimal value, the negative impact of viruses on the host (virulence) removes the host from being infectious, e.g. via host death or hospitalization, thereby decreasing the virus's transmissibility. In light of this, we postulate two potential mechanisms by which the tDP could modulate transmission. The first is that the tDP reduces viral loads through interference, as is known for some other defective particles [3]. Lower viral loads lead to milder disease [40], which allows dually infected humans to be more mobile than humans infected with wild-type DENV-1 only. Because the spatial spread of dengue is driven chiefly by human movements [32], dually infected humans can facilitate greater disease dissemination. This scenario is plausible if the virulence of the functional virus in humans exceeds the optimal value for transmission. The second possible mechanism is that the tDP increases production of the functional virus, by circumventing constraints in viral gene expression within a cell. Differential gene expression is a major challenge for (+)ssRNA viruses such as dengue, because of constraints arising from their genomic architecture and particularly the necessity to translate individual protein products from a single polyprotein precursor [41]. The presence of tDP in either infected human cells or mosquito cells could increase the abundance of gene products that are otherwise limiting, thereby increasing virus fitness. This scenario is plausible if the current dengue viral loads in either humans or mosquitoes are below the optimal value for transmission. While we cannot discriminate between these competing hypotheses with current data, they could be tested by measuring the relative viral load or clinical severity of dually infected versus singly infected hosts, and would yield interesting insights about the virulence of DENV-1.

The potential for the presence of tDPs to increase the transmission potential of DENV-1 suggests that tDPs may emerge and spread often, raising the question of why tDPs have not been reported more frequently and in more geographic regions. This could be explained by study designs that have focused almost exclusively on consensus sequences, thereby avoiding any dissection of intra-host genetic variation. In addition, our simulations (Fig. 3) suggest that higher transmission potential of the co-transmission route may cause the tDP to go extinct due to depletion of the susceptible population following epidemics. Hence, tDPs may have emerged and died out multiple times in history. Indeed, defective DENV-1 lineages harboring the same stop-codon mutation have been identified elsewhere on at least one occasion [19]. Finally, the conditions that favor tDP emergence may depend on local ecological or epidemiological factors, such as human movement patterns, vector species or strains, and immunological interactions between the four serotypes of dengue. More intensive sampling, and sequencing efforts focusing on intra-host dengue diversity, would help to characterize the true frequency of tDP emergence and spread in populations worldwide.

Our model predicts that the emergence of tDPs should lead to a substantial increase in DENV-1 incidence. This prediction arises wholly from our finding that co-transmission of tDP and DENV-1 is more efficient than wild-type transmission, which is derived only from the rise in relative frequency of dual infection and is robust across epidemiological backgrounds. Although limited data prevent a precise assessment of this prediction, it is consistent with the observation that the number of reported dengue cases reached historically high levels during the 2001 and 2002 seasons [18]. Of course, many other factors can influence dengue epidemiology, such as immunological interactions arising from switches in dominant serotypes [42] or changes of fitness resulting from mutations elsewhere in the viral genome [43]. However, these outbreaks do not share patterns typically associated with serotype switches, since all four dengue serotypes were circulating in Myanmar leading up to the large outbreak in 2001, and almost half of the DENV-1 infections in 2001 were primary infections [18]. It is also possible that an increase in reported incidence could be explained by improved surveillance, although this is unlikely given that a comprehensive clinical and laboratory surveillance program has been established in Myanmar since 1984 and did not change in the years when higher numbers of cases were reported.

The existence of tDPs for dengue virus raises the possibility that sustained transmission of defective particles may be a more general phenomenon for other viruses. Our analyses highlight several conditions that facilitate long-term spread of defective particles: 1) relatively wide transmission bottlenecks, 2) frequent dual infection at the level of hosts and the level of cells, and 3) potential to increase the transmissibility of the functional virus by modulating the viral load within hosts. Interestingly, a recent study provided evidence that a lineage of defective particles of canine influenza transmitted for at least 4-months in a high-density dog population [11]. Similarly, the transmission of defective particles (characterized by stop codon mutations) in experimental transmission studies of swine influenza among pigs has also been reported [44]. As sampling efforts focusing on within-host genetic diversity become more common, it is likely that tDPs will be observed more frequently than currently appreciated. This would narrow the functional distinction between defective particles and satellite viruses, another well-known class of transmissible sub-viral agents, which also require complementation but are not immediately derived from their helper viruses [17].

Finally, our work reveals some general principles concerning viral emergence. First, complementation can be a powerful factor in determining the evolutionary dynamics of natural viral populations. The extreme case reported here, with long-term spread of a totally defective viral lineage, has implications for emergence pathways that need to cross fitness valleys [45]. If co-infection is common, a lineage could easily cross a wide fitness valley by pairing with a competent strain. Second, interactions among strains that lead to modified virulence (or other host-level phenotypes) can lead to increased transmission fitness [23], [46], and hence emergence. Altogether, this case study expands the range of mechanisms that may be pertinent to the study of viral emergence, and re-emphasizes the need to use appropriate models, at intra- and inter-host scales, to understand the processes giving rise to epidemiological patterns and associated pathogen sequence data.

## Methods

### Phylogenetic analysis

The 290 sequences and the eight additional sequences from Myanmar were extracted from Genbank (accession numbers DQ264868 to DQ265157, AY588273, AY606062, AY618877, AY618878, AY618880, AY620948, AY620950 and AY726555). The 24 additional sequences from the patient in New Caledonia were obtained from Ref. [13]. The relationship of these 24 sequences with the sequences from Myanmar was evaluated by constructing a phylogenetic tree using the maximum likelihood method in Garli 0.951 [47], employing the GTR+I+Γ_4_ model of nucleotide substitution. The ratio of nonsynonymous to synonymous substitutions per site (d_N_/d_S_) was estimated using the Datamonkey webserver [48] employing the SLAC method [49]. The time of tDP emergence was estimated by constructing a Maximum Clade Credibility (MCC) tree using BEAST, again employing the GTR+I+Γ_4_ model [33]. The time to common ancestry was estimated using the Bayesian skyline coalescent model and an uncorrelated lognormal relaxed clock model [50], with a total of 5,000,000 states collected from the MCMC chain and the first 500,000 states excluded as burn-in. The effective sample size for each parameter in the estimation was checked using Tracer v1.5 to ensure convergence [51], with statistical uncertainty reflected in values of the 95% Highest Probability Density (HPD). Note that the exact months when the additional eight sequences [18] were isolated is unknown. We assumed that they were isolated in June of the appropriate year, and confirmed that estimation of the time of tDP emergence was robust to the choice of month of isolation. Nucleotide sequences and relevant parameters estimated in the software are available from authors upon request. Tree topology tests were performed using TREE-PUZZLE 5.2 with HKY+Γ_4_ [52].

### Mathematical model for transmission dynamics

We constructed a human-vector SEIR compartmental model considering the dynamics of DENV-1 and tDP (schematic shown in Fig. 2C). This model considers the demographic changes of human and mosquito populations, with the mosquito birth rate seasonally forced in accordance with monthly data [53]. It keeps track of the infection dynamics of one dengue serotype (DENV-1) and its associated defective particles (tDP) at the population scale. The full model is shown in Eqn. 1. The human population size (N_H_) is assumed to be constant, with individuals born into the susceptible compartment (S_H_) at *per capita* rate *μ_H_*, and all human individuals subject to *per capita* death rate *μ_H_*. The rate constant for transmission of DENV-1, encompassing the contact rate and probability of transmission, is *β*. When susceptible humans have contact with dually infected mosquitoes, three types of transmission events can potentially occur (Fig. 2A,B): tDP transmission, DENV-1 transmission and dual transmission. The three scaling parameters, *P, Q* and *W*, are used to model the efficiency of these three types of transmission, respectively, relative to the transmission rate (*β*) from individuals infected only with DENV-1. We assume that dually infected individuals may have different infection characteristics from DENV-1 infected individuals, and hence may differ in the latent period, infectious period and recovery rate. The mean latent periods of DENV-1 infected individuals and dually infected individuals are *1/σ_H_* and *1/σ_H,D_*, respectively. The DENV-1 infected latent (E) or infectious (I) individual can move to the dually infected latent (G) or infectious (D) compartment if they are super-infected by tDP. The infectious DENV-1 infected individuals (I) and dually infected individuals (D) recover to become recovered (and immune) individuals (R) at rates *γ_0_* and *γ_1_*, respectively.

For the mosquito population, we do not consider vertical transmission of dengue virus, since it has been shown that vertical transmission at the rates reported in the literature does not have a strong impact on transmission dynamics [54]. We explicitly consider the seasonal forcing (a*cos(2*π*t+b)) of the mosquito birth rate due to changes in rainfall and temperature. The infection dynamics for the mosquito are modeled in a similar manner to the human infection dynamics, except that mosquitoes do not recover from dengue infection. State variables for the mosquito population match those for the human population, with an additional subscript ‘V’.

The resulting ordinary differential equation model is shown below:
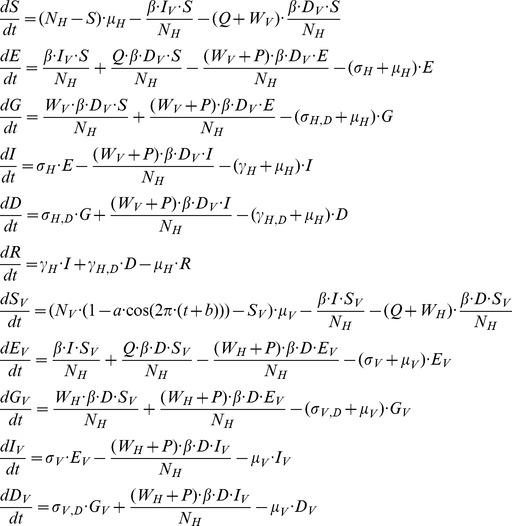
(1)


The description and initial values of the state variables are shown in Table S6, and the description and the values of the parameters in the equation are shown in Table S7.

We do not consider other dengue serotypes and their immunological effects on DENV-1 transmission in our model. This is because DENV-1 was the major circulating strain from 2000 to 2002 in Myanmar, and almost half of the DENV-1 infections in 2001 were primary infections [18], indicating that serotype interactions were not the dominant driver of observed dynamics. Importantly, tDP has been associated with DENV-1 only, and thus it experiences the same competitive interactions with other serotypes as wild-type DENV-1. Therefore, the increased frequency of dually infected individuals among DENV-1 infected individuals (which is the primary basis for our model conclusions) should be independent of the interactions with other serotypes. Hence, this simplification of the model will not alter the major findings of our study.

The model was first simulated without tDPs to establish the baseline endemic dynamics of dengue in the population. Based on the observation that DENV-1 incidence peaked in 2001 and 2002 in Myanmar, we defined two consecutive years with peak incidences of dengue to be 2001 and 2002 in the simulation. Using this simulation scenario as a baseline, tDP was introduced into the system at the beginning of year 2000, and the model was simulated another 3 years after tDP introduction to generate the results for Figs. 3 and 4. The qualitative conclusion that the presence of tDP increases DENV-1 transmission is robust to the choice of this mapping between simulation and calendar years, as long as dengue is endemic in the model (Fig. S3 and Table S5), but quantitative predictions of the magnitude of the rise in DENV-1 incidence differ. The details of the sensitivity analyses and the likelihood-based procedure for estimation of R_eff,co_ and *t_emg_* are presented in Text S1.

### Reproductive number for co-transmission of tDP and DENV-1

We performed a next-generation matrix analysis [55] on the simplified model, to calculate the effective reproduction number, R_eff,co_, for the co-transmission route. R_eff,co_ is defined as the average number of secondary dually infected cases infected through the co-transmission route by the first dually infected individual, when it is introduced into the system where the number of DENV-1-only infected cases is at non-zero equilibrium. Then, the condition for tDP emergence is R_eff,co_>1. Given our emphasis on the phenomenon of co-transmission, we further distinguish co-transmission from human-to-mosquito and mosquito-to-human using parameters *W_H_* and *W_V_*, respectively. We also allow for the possibility that dually infected human and mosquito individuals might have incubation periods and infectious periods that differ from singly infected individuals. Then, R_eff,co_ can be approximated as:
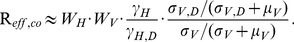
(2)


Therefore, the emergence of the tDP is determined by four parameters characterizing the dually infected individuals: the scaling parameters for force of infection, *W_H_*, *W_V_*, the infectious period of dually infected humans, 1/*γ_H,D_* and the incubation period of dually infected mosquitoes,1/*σ_V,D_*.

## Supporting Information

Figure S1 **Three tree topologies tested for the evolutionary history of the wt-1 lineage sequence from individual 47662 (47662_1 in red).** Only subsets of the three tree topologies are shown. The topology of Tree N is extracted from the phylogenetic tree in Fig. 1B. In Tree A and B, 47662_1 is assumed to be the descendant of the founding genome. The likelihood scores for the three trees are shown in Table S2.
(TIF)

Figure S2 **Simulations with parameter values estimated using maximum likelihood estimation and comparisons with data.** (**A**) Simulated numbers of total DENV-1 cases over time in a model without tDP emergence (dashed black line) and models using maximum likelihood parameter values as shown in Table 1. The simulations for the four scenarios analyzed in the main text, denoted as ‘W_H_’, ‘W_V_’, ‘γ_H,D_’ and ‘σ_V,D_’, are shown as blue, red, green and cyan lines, respectively. (**B**) The frequency of dually infected among all infected human individuals in corresponding simulations shown in panel A. (C) The simulated and reported frequencies of dually infected mosquitoes and humans in year 2001 and 2002. The simulated frequencies were obtained from the simulation shown in panel A. The reported frequency calculated using the data reported by Aaskov et al. [14]. The error bars show the 95% confidence intervals calculated assuming binomial sampling.
(TIF)

Figure S3 **Results of maximum likelihood estimations are robust across different phases of background DENV-1 dynamics.** The mappings we considered between simulation year (t_2001_) and the calendar year 2001 are t_2001_ = 46, 48, 54 and 64. The simulations using parameter values estimated from MLE (Table S5) and their comparisons with data are shown in panels (A,E,I), panels (B,F,J), panels (C,G,K) and panels (D,H,L) for the four schemes, respectively. The figure legends follow the same notation as Fig. S2.
(TIF)

Table S1 **Intra- and inter- host genetic diversity for the stop-codon lineage and the std-1 lineage.** For the intra-host genetic diversity, the average number of pairwise segregation sites (π) and the ratios of the numbers of non-synonymous over synonymous mutations (d_N_/d_S_) in each individual are examined for the stop-codon and the wt-1 lineage. For the inter-host genetic diversity, the nucleotide distances between the consensus sequences of each lineage in each individual and the reference sequence of the corresponding lineage are calculated. The reference sequence of a lineage is defined as the consensus sequence shared by the greatest number of individuals in that lineage.
(PDF)

Table S2 **Topology test results for 3 phylogenetic trees shown Fig. S1.**
(PDF)

Table S3 **Maximum-likelihood estimates (MLE) of parameter values and their corresponding increase in overall DENV-1 cases during 2001 and 2002.** Seasonal forcing parameter *a* = 0.6.
(PDF)

Table S4 **Maximum-likelihood estimates of parameter values and their corresponding increase in overall DENV-1 cases during 2001 and 2002.** Seasonal forcing parameter *a* = 0.8.
(PDF)

Table S5 **Results of maximum likelihood estimations are robust across different phases of background DENV-1 dynamics.** The values of maximum likelihood estimations are shown. The corresponding simulations are shown in Fig. S3.
(PDF)

Table S6 **Description and initial values of the state variables of the ODE model.**
(PDF)

Table S7 **Parameter values of the full ODE model.**
(PDF)

Text S1 **Supporting Text.**
(PDF)
