# Supplementary figures and images for: Phylodynamic Analysis of the Emergence and Epidemiological Impact of Transmissible Defective Dengue Viruses
Source: PLoS Pathog. 2013 Feb 28;9(2):e1003193. doi: 10.1371/journal.ppat.1003193 (PMC3585136; doi:10.1371/journal.ppat.1003193)

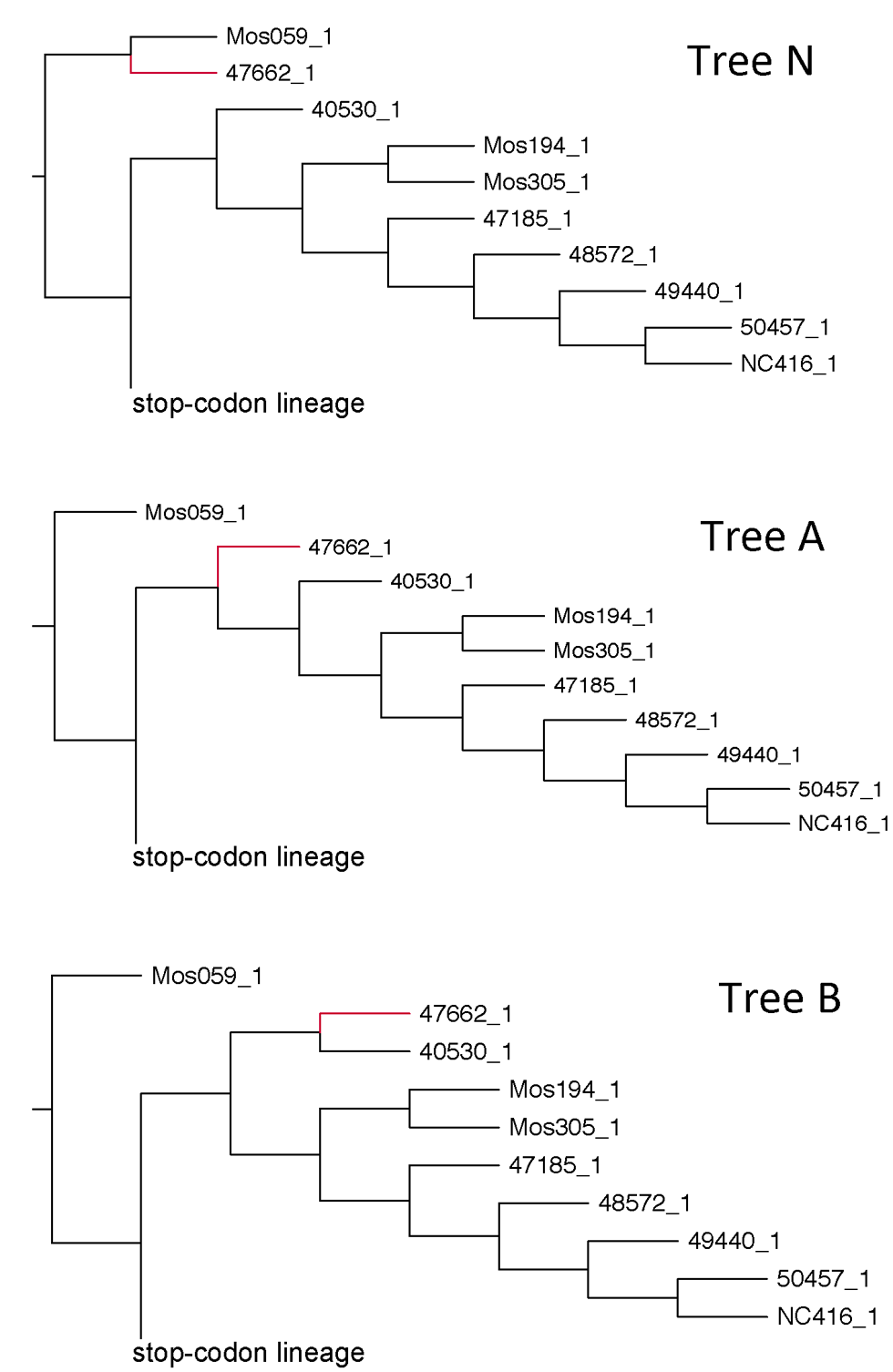

Supplement: Figure S1 — Three tree topologies tested for the evolutionary history of the wt-1 lineage sequence from individual 47662 (47662_1 in red). Only subsets of the three tree topologies are shown. The topology of Tree N is extracted from the phylogenetic tree in Fig. 1B. In Tree A and B, 47662_1 is assumed to be the descendant of the founding genome. The likelihood scores for the three trees are shown in Table S2. (TIF) [file ppat.1003193.s001.tif]

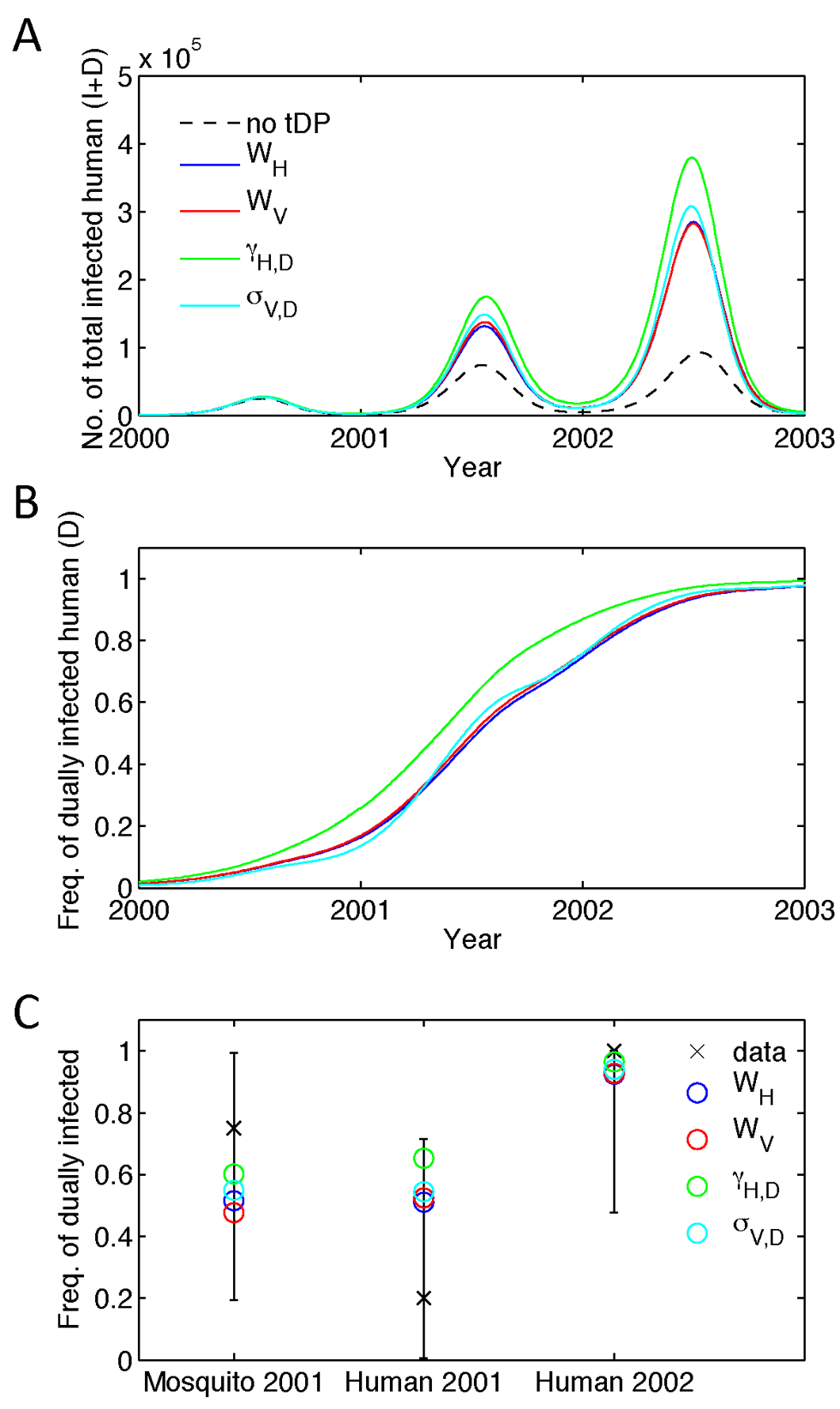

Supplement: Figure S2 — Simulations with parameter values estimated using maximum likelihood estimation and comparisons with data. (A) Simulated numbers of total DENV-1 cases over time in a model without tDP emergence (dashed black line) and models using maximum likelihood parameter values as shown in Table 1. The simulations for the four scenarios analyzed in the main text, denoted as ‘WH’, ‘WV’, ‘γH,D’ and ‘σV,D’, are shown as blue, red, green and cyan lines, respectively. (B) The frequency of dually infected among all infected human individuals in corresponding simulations shown in panel A. (C) The simulated and reported frequencies of dually infected mosquitoes and humans in year 2001 and 2002. The simulated frequencies were obtained from the simulation shown in panel A. The reported frequency calculated using the data reported by Aaskov et al. [14]. The error bars show the 95% confidence intervals calculated assuming binomial sampling. (TIF) [file ppat.1003193.s002.tif]

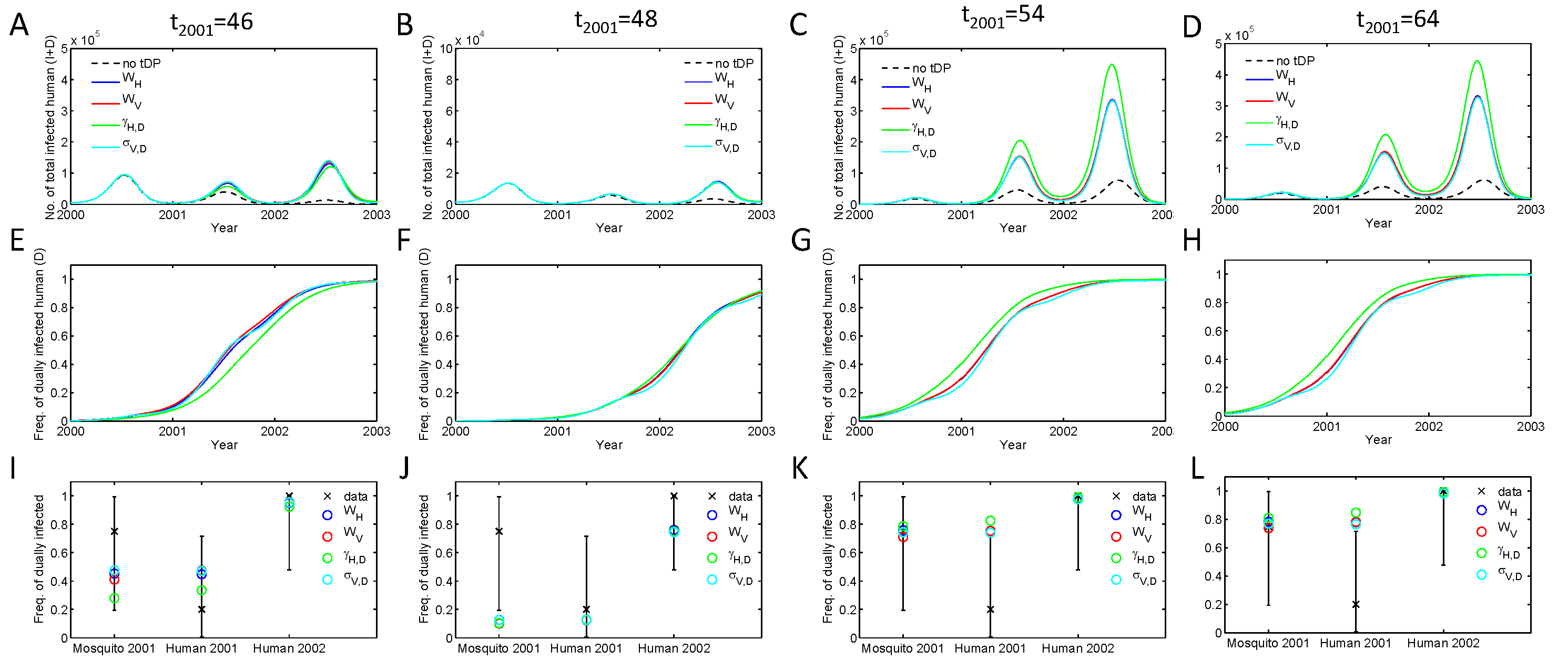

Supplement: Figure S3 — Results of maximum likelihood estimations are robust across different phases of background DENV-1 dynamics. The mappings we considered between simulation year (t2001) and the calendar year 2001 are t2001 = 46, 48, 54 and 64. The simulations using parameter values estimated from MLE (Table S5) and their comparisons with data are shown in panels (A,E,I), panels (B,F,J), panels (C,G,K) and panels (D,H,L) for the four schemes, respectively. The figure legends follow the same notation as Fig. S2. (TIF) [file ppat.1003193.s003.tif]
