# Supplementary material for: Phylodynamic Analysis of the Emergence and Epidemiological Impact of Transmissible Defective Dengue Viruses
Source: PLoS Pathog. 2013 Feb 28;9(2):e1003193. doi: 10.1371/journal.ppat.1003193 (PMC3585136; doi:10.1371/journal.ppat.1003193)
Supplement: Table S1 — Intra- and inter- host genetic diversity for the stop-codon lineage and the std-1 lineage. For the intra-host genetic diversity, the average number of pairwise segregation sites (π) and the ratios of the numbers of non-synonymous over synonymous mutations (dN/dS) in each individual are examined for the stop-codon and the wt-1 lineage. For the inter-host genetic diversity, the nucleotide distances between the consensus sequences of each lineage in each individual and the reference sequence of the corresponding lineage are calculated. The reference sequence of a lineage is defined as the consensus sequence shared by the greatest number of individuals in that lineage. (PDF) [file ppat.1003193.s004.pdf]

Table S1. Intra- and inter- host genetic diversity for the stop-codon lineage and the std-1 lineage. For the intra-host genetic diversity, the average number of pairwise segregation sites (π) and the ratios of the numbers of non-synonymous over synonymous mutations (d_N/d_S) in each individual are examined for the stop-codon and the wt-1 lineage. For the inter-host genetic diversity, the nucleotide distances between the consensus sequences of each lineage in each individual and the reference sequence of the corresponding lineage are calculated. The reference sequence of a lineage is defined as the consensus sequence shared by the greatest number of individuals in that lineage.

		Stop codon lineage					wt-1 lineage			
	ID	No. clones	π	d_N/d_S [a] 5' to Stop	d_N/d_S [a] Stop to 3'	Nucleotide distance to the Ref. Seq.	No. clones	π	d_N/d_S [a]	Nucleotide distance to the Ref. Seq.
Individuals with tDIP	*Mos194*	10	20.8	0.631	1.379	1	10	19.3	0.658	0
	Mos206	6	0.6	--	--	0	1	--	--	--
	Mos305	10	11.9	0.827	0.642	0	10	8.9	0.831	0
	40530	1	--	--	--	--	18	8.9	0.691	1
	47185	9	10.4	1.713	1.005	1	11	11.8	0.447	0
	47662	1	--	--	--	--	19	10.4	0.651	2
	48572	11	7.4	0.595	1.018	0	9	4.5	0.996	0
	49440	11	5.9	0.949	0.515	0	9	14.9	0.351	0
	50457	10	11.9	0.547	1.863	0	10	16.3	0.456	0
	NC416	7	20.8	1.931	1.251	5	4	20.8	0.864	0-4[b]
Individuals without tDIP	*Mos059*	0	--	--	--	--	20	14.8	0.845	4
	37045	0	--	--	--	--	20	16.3	0.685	9
	38518	0	--	--	--	--	19	14.9	0.592	6
	40906	0	--	--	--	--	20	11.9	0.593	8
	43549	0	--	--	--	--	20	5.9	0.985	7

[a] d_N/d_S is calculated on the Datamonkey webserver (2) using the SLAC method (3).

[b] The consensus for 4 nucleotides of the sequences in this lineage cannot be determined.
