# Supplementary material for: Phylodynamic Analysis of the Emergence and Epidemiological Impact of Transmissible Defective Dengue Viruses
Source: PLoS Pathog. 2013 Feb 28;9(2):e1003193. doi: 10.1371/journal.ppat.1003193 (PMC3585136; doi:10.1371/journal.ppat.1003193)
Supplement: Table S2 — Topology test results for 3 phylogenetic trees shown Fig. S1. (PDF) [file ppat.1003193.s005.pdf]

Table S2. Topology test results[a] for 3 phylogenetic trees shown Fig.S1.

Tree ID	Log Likelihood	p-values of S.H. test	E.L.W.
**N**	-2955.47	1.0000	0.7936
**A**	-2960.01	0.1270	0.1489
**B**	-2962.77	0.0950	0.0574

[a] All tests used 5% significance level. 1000 resamples were performed in Shimodaira-Hasegawa (S.H.) test (4), and the Expected Likelihood Weight (E.L.W.) test (5) using the RELL method (6).
