# Supplementary material for: Phylodynamic Analysis of the Emergence and Epidemiological Impact of Transmissible Defective Dengue Viruses
Source: PLoS Pathog. 2013 Feb 28;9(2):e1003193. doi: 10.1371/journal.ppat.1003193 (PMC3585136; doi:10.1371/journal.ppat.1003193)
Supplement: Table S3 — Maximum-likelihood estimates (MLE) of parameter values and their corresponding increase in overall DENV-1 cases during 2001 and 2002. Seasonal forcing parameter a = 0.6. (PDF) [file ppat.1003193.s006.pdf]

Table S3. Maximum-likelihood estimates (MLE) of parameter values and their corresponding increase in overall DENV-1 cases during 2001 and 2002. Seasonal forcing parameter *α*=0.6.

Parameter	Value (C.I. [a])	$R_{eff,co}$	t_{emg}	Neg. Log-Likelihood	Fold increase in DENV-1 cases during 2001-2002 (range of variation [b])
W_H	1.25 (1.13-1.75)	1.25 (1.13-1.75)	Nov, 1998	4.97	2.3 (1.4-3.3)
W_V	1.25 (1.14-1.80)	1.25 (1.14-1.80)	Dec, 1998	5.28	2.3 (1.5-3.4)
$\gamma_{H,D}$	0.129 day^{-1} (0.10-0.15)	1.29 (1.15-1.61)	Dec, 1998	5.67	3.1 (1.7-4.0)
$\sigma_{V,D}$	0.189 day^{-1} (0.14-0.79)	1.24 (1.13-1.57)	Feb, 1999	4.88	2.4 (1.5-3.2)

[a] 95% confidence intervals that are calculated using parameter profiling.

[b] The ranges of variation are calculated using the estimated parameter values at the boundaries of the 95% confidence intervals.
