# Supplementary material for: Phylodynamic Analysis of the Emergence and Epidemiological Impact of Transmissible Defective Dengue Viruses
Source: PLoS Pathog. 2013 Feb 28;9(2):e1003193. doi: 10.1371/journal.ppat.1003193 (PMC3585136; doi:10.1371/journal.ppat.1003193)
Supplement: Table S4 — Maximum-likelihood estimates of parameter values and their corresponding increase in overall DENV-1 cases during 2001 and 2002. Seasonal forcing parameter a = 0.8. (PDF) [file ppat.1003193.s007.pdf]

Table S1. Intra- and inter- host genetic diversity for the stop-codon lineage and the std-1 lineage. For the intra-host genetic diversity, the average number of pairwise segregation sites (π) and the ratios of the numbers of non-synonymous over synonymous mutations (d_N/d_S) in each individual are examined for the stop-codon and the wt-1 lineage. For the inter-host genetic diversity, the nucleotide distances between the consensus sequences of each lineage in each individual and the reference sequence of the corresponding lineage are calculated. The reference sequence of a lineage is defined as the consensus sequence shared by the greatest number of individuals in that lineage.

		Stop codon lineage					wt-1 lineage			
	ID	No. clones	π	d_N/d_S [a] 5' to Stop	d_N/d_S [a] Stop to 3'	Nucleotide distance to the Ref. Seq.	No. clones	π	d_N/d_S [a]	Nucleotide distance to the Ref. Seq.
Individuals with tDIP	*Mos194*	10	20.8	0.631	1.379	1	10	19.3	0.658	0
	Mos206	6	0.6	--	--	0	1	--	--	--
	Mos305	10	11.9	0.827	0.642	0	10	8.9	0.831	0
	40530	1	--	--	--	--	18	8.9	0.691	1
	47185	9	10.4	1.713	1.005	1	11	11.8	0.447	0
	47662	1	--	--	--	--	19	10.4	0.651	2
	48572	11	7.4	0.595	1.018	0	9	4.5	0.996	0
	49440	11	5.9	0.949	0.515	0	9	14.9	0.351	0
	50457	10	11.9	0.547	1.863	0	10	16.3	0.456	0
	NC416	7	20.8	1.931	1.251	5	4	20.8	0.864	0-4[b]
Individuals without tDIP	*Mos059*	0	--	--	--	--	20	14.8	0.845	4
	37045	0	--	--	--	--	20	16.3	0.685	9
	38518	0	--	--	--	--	19	14.9	0.592	6
	40906	0	--	--	--	--	20	11.9	0.593	8
	43549	0	--	--	--	--	20	5.9	0.985	7

[a] d_N/d_S is calculated on the Datamonkey webserver (2) using the SLAC method (3).

[b] The consensus for 4 nucleotides of the sequences in this lineage cannot be determined.

Table S2. Topology test results[a] for 3 phylogenetic trees shown Fig.S1.

Tree ID	Log Likelihood	p-values of S.H. test	E.L.W.
**N**	-2955.47	1.0000	0.7936
**A**	-2960.01	0.1270	0.1489
**B**	-2962.77	0.0950	0.0574

[a] All tests used 5% significance level. 1000 resamples were performed in Shimodaira-Hasegawa (S.H.) test (4), and the Expected Likelihood Weight (E.L.W.) test (5) using the RELL method (6).

Table S3. Maximum-likelihood estimates (MLE) of parameter values and their corresponding increase in overall DENV-1 cases during 2001 and 2002. Seasonal forcing parameter *α*=0.6.

Parameter	Value (C.I. [a])	$R_{eff,co}$	t_{emg}	Neg. Log-Likelihood	Fold increase in DENV-1 cases during 2001-2002 (range of variation [b])
W_H	1.25 (1.13-1.75)	1.25 (1.13-1.75)	Nov, 1998	4.97	2.3 (1.4-3.3)
W_V	1.25 (1.14-1.80)	1.25 (1.14-1.80)	Dec, 1998	5.28	2.3 (1.5-3.4)
$\gamma_{H,D}$	0.129 day^{-1} (0.10-0.15)	1.29 (1.15-1.61)	Dec, 1998	5.67	3.1 (1.7-4.0)
$\sigma_{V,D}$	0.189 day^{-1} (0.14-0.79)	1.24 (1.13-1.57)	Feb, 1999	4.88	2.4 (1.5-3.2)

[a] 95% confidence intervals that are calculated using parameter profiling.

[b] The ranges of variation are calculated using the estimated parameter values at the boundaries of the 95% confidence intervals.

Table S4. Maximum-likelihood estimates of parameter values and their corresponding increase in overall DENV-1 cases during 2001 and 2002. Seasonal forcing parameter *a*=0.8.

Parameter	Value (C.I. [a])	$R_{eff,co}$	t_{emg}	Neg. Log-Likelihood	Fold increase in DENV-1 cases during 2001-2002 (range of variation [b])
W_H	1.23 (1.12-1.66)	1.23 (1.12-1.66)	Dec, 1998	5.22	2.5 (1.4-3.4)
W_V	1.23 (1.13-1.60)	1.23 (1.13-1.60)	Dec, 1998	5.39	2.5 (1.5-3.3)
$\gamma_{H,D}$	0.132 day^{-1} (0.11-0.15)	1.27 (1.12-1.48)	Dec, 1998	6.26	3.3 (1.5-4.1)
$\sigma_{V,D}$	0.164 day^{-1} (0.13-0.75)	1.19 (1.12-1.57)	Dec, 1998	4.89	2.3 (1.4-3.3)

[a] 95% confidence intervals that are calculated using parameter profiling.

[b] The ranges of variation are calculated using the estimated parameter values at the boundaries of the 95% confidence intervals.

Table S5. Results of maximum likelihood estimations are robust across different phases of background DENV-1 dynamics. The values of maximum likelihood estimations are shown. The corresponding simulations are shown in Fig. S3.

Parameter	Simulation time for year 2001 (t_{2001})	Maximum Likelihood estimate	$R_{eff,co}$	Neg. Log-Likelihood	Fold increase in DENV-1 cases during 2001-2002
W_H	44	1.23	1.23	4.99	2.3
	46	1.32	1.32	4.92	3.4
	48	1.29	1.29	8.74	3.4
	54	1.29	1.29	6.70	3.7
	64	1.31	1.31	7.11	4.3
	74	1.32	1.32	7.06	4.6
W_V	44	1.23	1.23	5.26	2.4
	46	1.32	1.32	5.31	3.6
	48	1.29	1.29	9.32	3.6
	54	1.28	1.28	6.76	3.7
	64	1.31	1.31	7.18	4.3
	74	1.32	1.32	7.05	4.5
$\gamma_{H,D}$	44	0.131 day^{-1}	1.28	5.81	3.2
	46	0.126	1.33	5.79	3.2
	48	0.128	1.30	9.32	3.2
	54	0.125 day^{-1}	1.33	8.09	5.1
	64	0.123 day^{-1}	1.35	8.63	6.0
	74	0.122 day^{-1}	1.36	8.55	6.4
$\sigma_{V,D}$	44	0.186 day^{-1}	1.24	4.88	2.5
	46	0.223	1.30	4.75	3.5
	48	0.198	1.26	8.39	3.5
	54	0.195 day^{-1}	1.25	6.46	3.5
	64	0.203 day^{-1}	1.27	6.81	4.1
	74	0.206 day^{-1}	1.27	6.65	4.2

Table S6. Description and initial values of the state variables of the ODE model.

State variables	Description	Initial value*
**S**	Number of susceptible humans	$0.3*N_H$
**E**	Number of exposed humans who are infected with DENV-1	0
**I**	Number of infectious humans who are infected with DENV-1	1
**R**	Number of recovered humans	$0.7*N_H$
**T**	Number of humans infected with tDP only	0
**G**	Number of exposed humans who are infected with both DENV-1 and tDP	0
**D**	Number of infectious humans who are infected with both DENV-1 and tDP	0
$\mathbf{S_V}$	Number of susceptible mosquitoes	N_V
$\mathbf{E_V}$	Number of exposed mosquitoes infected with DENV-1	0
$\mathbf{I_V}$	Number of infectious mosquitoes infected with DENV-1	0
$\mathbf{T_V}$	Number of mosquitoes infected with tDP only	0
$\mathbf{G_V}$	Number of exposed mosquitoes infected with both DENV-1 and tDP	0
$\mathbf{D_V}$	Number of Infectious mosquitoes infected with both DENV-1 and tDP	0

* N_H and N_V are the total numbers of humans and mosquitoes, respectively (see Table S6).

Table S7. Parameter values of the full ODE model.

Parameter	Description	Value	Ref
N_H	Human population in Myanmar	50,000,000	a
N_V	Mosquito population in Myanmar	N_H*2	[2]
β	Transmission rate	70 $year^{-1}$	[2]
μ_H	Human host birth rate = death rate	1/60 $year^{-1}$	[2]
μ_V	Mosquito birth rate = death rate	365/14 $year^{-1}$	[2]
$1/\sigma_H$	Average latent period of human infected with DENV-1 only	5/365 year	[2]
$1/\sigma_{H,D}$	Average latent period of dually infected human	Varies in model	
$1/\sigma_V$	Average incubation period of mosquitoes infected with DENV-1 only	10/365 year	[2]
$1/\sigma_{V,D}$	Average incubation period of dually infected mosquitoes	Varies in model	
$1/\gamma_H$	Average infectious period of human infected with DENV-1 only	6/365 year	[2]
$1/\gamma_{H,D}$	Average infectious period of dually infected human	Varies in model	
**a**	Seasonality coefficient for mosquito birth rate	0.7 (unless specified)	b
**b**	Phase of seasonality. The value of b is chosen such that the number of dengue cases peaks in July as shown in [3].	0.3	b
**P**	Relative efficiency of tDP-only transmission from dually infected individuals compared to the rate of DENV-1 transmission from singly infected individuals	Varies in model<br>Baseline value: 0.01	
**Q**	Relative efficiency of DENV-1-only transmission from dually infected individuals compared to the rate of DENV-1 transmission from singly infected individuals	Varies in model<br>Baseline value: 0.01	
**W**	Relative efficiency of dual transmission from dually infected individuals compared to the rate of DENV-1 transmission from singly infected individuals. When the dually infected humans and mosquitoes are considered separately, W_H and W_V denote the relative rates of dual transmission from dually infected humans and mosquitoes, respectively.	Varies in model<br>Baseline value: 1.00	

[a] Source: Myanmar 2000,United Nations.

[b] The seasonality parameters are chosen such that the seasonal pattern in the model simulation is consistent with the monthly case reports in [3].
