# Supplementary material for: Phylodynamic Analysis of the Emergence and Epidemiological Impact of Transmissible Defective Dengue Viruses
Source: PLoS Pathog. 2013 Feb 28;9(2):e1003193. doi: 10.1371/journal.ppat.1003193 (PMC3585136; doi:10.1371/journal.ppat.1003193)
Supplement: Table S5 — Results of maximum likelihood estimations are robust across different phases of background DENV-1 dynamics. The values of maximum likelihood estimations are shown. The corresponding simulations are shown in Fig. S3. (PDF) [file ppat.1003193.s008.pdf]

Table S5. Results of maximum likelihood estimations are robust across different phases of background DENV-1 dynamics. The values of maximum likelihood estimations are shown. The corresponding simulations are shown in Fig. S3.

Parameter	Simulation time for year 2001 (t_{2001})	Maximum Likelihood estimate	$R_{eff,co}$	Neg. Log-Likelihood	Fold increase in DENV-1 cases during 2001-2002
W_H	44	1.23	1.23	4.99	2.3
	46	1.32	1.32	4.92	3.4
	48	1.29	1.29	8.74	3.4
	54	1.29	1.29	6.70	3.7
	64	1.31	1.31	7.11	4.3
	74	1.32	1.32	7.06	4.6
W_V	44	1.23	1.23	5.26	2.4
	46	1.32	1.32	5.31	3.6
	48	1.29	1.29	9.32	3.6
	54	1.28	1.28	6.76	3.7
	64	1.31	1.31	7.18	4.3
	74	1.32	1.32	7.05	4.5
$\gamma_{H,D}$	44	0.131 day^{-1}	1.28	5.81	3.2
	46	0.126	1.33	5.79	3.2
	48	0.128	1.30	9.32	3.2
	54	0.125 day^{-1}	1.33	8.09	5.1
	64	0.123 day^{-1}	1.35	8.63	6.0
	74	0.122 day^{-1}	1.36	8.55	6.4
$\sigma_{V,D}$	44	0.186 day^{-1}	1.24	4.88	2.5
	46	0.223	1.30	4.75	3.5
	48	0.198	1.26	8.39	3.5
	54	0.195 day^{-1}	1.25	6.46	3.5
	64	0.203 day^{-1}	1.27	6.81	4.1
	74	0.206 day^{-1}	1.27	6.65	4.2
