# Supplementary material for: Phylodynamic Analysis of the Emergence and Epidemiological Impact of Transmissible Defective Dengue Viruses
Source: PLoS Pathog. 2013 Feb 28;9(2):e1003193. doi: 10.1371/journal.ppat.1003193 (PMC3585136; doi:10.1371/journal.ppat.1003193)
Supplement: Table S6 — Description and initial values of the state variables of the ODE model. (PDF) [file ppat.1003193.s009.pdf]

Table S6. Description and initial values of the state variables of the ODE model.

State variables	Description	Initial value*
**S**	Number of susceptible humans	$0.3*N_H$
**E**	Number of exposed humans who are infected with DENV-1	0
**I**	Number of infectious humans who are infected with DENV-1	1
**R**	Number of recovered humans	$0.7*N_H$
**T**	Number of humans infected with tDP only	0
**G**	Number of exposed humans who are infected with both DENV-1 and tDP	0
**D**	Number of infectious humans who are infected with both DENV-1 and tDP	0
$\mathbf{S_V}$	Number of susceptible mosquitoes	N_V
$\mathbf{E_V}$	Number of exposed mosquitoes infected with DENV-1	0
$\mathbf{I_V}$	Number of infectious mosquitoes infected with DENV-1	0
$\mathbf{T_V}$	Number of mosquitoes infected with tDP only	0
$\mathbf{G_V}$	Number of exposed mosquitoes infected with both DENV-1 and tDP	0
$\mathbf{D_V}$	Number of Infectious mosquitoes infected with both DENV-1 and tDP	0

* N_H and N_V are the total numbers of humans and mosquitoes, respectively (see Table S6).
