# Supplementary material for: Phylodynamic Analysis of the Emergence and Epidemiological Impact of Transmissible Defective Dengue Viruses
Source: PLoS Pathog. 2013 Feb 28;9(2):e1003193. doi: 10.1371/journal.ppat.1003193 (PMC3585136; doi:10.1371/journal.ppat.1003193)
Supplement: Table S7 — Parameter values of the full ODE model. (PDF) [file ppat.1003193.s010.pdf]

Table S7. Parameter values of the full ODE model.

Parameter	Description	Value	Ref
N_H	Human population in Myanmar	50,000,000	[a]
N_V	Mosquito population in Myanmar	N_H*2	[2]
β	Transmission rate	70 $year^{-1}$	[2]
μ_H	Human host birth rate = death rate	1/60 $year^{-1}$	[2]
μ_V	Mosquito birth rate = death rate	365/14 $year^{-1}$	[2]
$1/\sigma_H$	Average latent period of human infected with DENV-1 only	5/365 year	[2]
$1/\sigma_{H,D}$	Average latent period of dually infected human	Varies in model	
$1/\sigma_V$	Average incubation period of mosquitoes infected with DENV-1 only	10/365 year	[2]
$1/\sigma_{V,D}$	Average incubation period of dually infected mosquitoes	Varies in model	
$1/\gamma_H$	Average infectious period of human infected with DENV-1 only	6/365 year	[2]
$1/\gamma_{H,D}$	Average infectious period of dually infected human	Varies in model	
**a**	Seasonality coefficient for mosquito birth rate	0.7 (unless specified)	[b]
**b**	Phase of seasonality. The value of b is chosen such that the number of dengue cases peaks in July as shown in [3].	0.3	[b]
**P**	Relative efficiency of tDP-only transmission from dually infected individuals compared to the rate of DENV-1 transmission from singly infected individuals	Varies in model<br>Baseline value: 0.01	
**Q**	Relative efficiency of DENV-1-only transmission from dually infected individuals compared to the rate of DENV-1 transmission from singly infected individuals	Varies in model<br>Baseline value: 0.01	
**W**	Relative efficiency of dual transmission from dually infected individuals compared to the rate of DENV-1 transmission from singly infected individuals. When the dually infected humans and mosquitoes are considered separately, W_H and W_V denote the relative rates of dual transmission from dually infected humans and mosquitoes, respectively.	Varies in model<br>Baseline value: 1.00	

[a] Source: Myanmar 2000,United Nations.
[b] The seasonality parameters are chosen such that the seasonal pattern in the model simulation is consistent with the monthly case reports in [3].
