# Supplementary material for: Phylodynamic Analysis of the Emergence and Epidemiological Impact of Transmissible Defective Dengue Viruses
Source: PLoS Pathog. 2013 Feb 28;9(2):e1003193. doi: 10.1371/journal.ppat.1003193 (PMC3585136; doi:10.1371/journal.ppat.1003193)
Supplement: Text S1 — Supporting Text. (PDF) [file ppat.1003193.s011.pdf]

Supporting Text (Text S1) for

'Phylodynamic analysis of the emergence and epidemiological impact of transmissible defective dengue viruses.'

Authors

Ruian Ke[a,1], John Aaskov[b], Edward C. Holmes[c,d] and James O. Lloyd-Smith[a,d,1]

Affiliations

[a]Department of Ecology and Evolutionary Biology, University of California, Los Angeles, 610 Charles E. Young Dr. South, Los Angeles, CA 90095, USA

[b]Institute of Health and Biomedical Innovation, Queensland University of Technology, 60 Musk Avenue, Brisbane, 4059, Australia

[c]School of Biological Sciences and Sydney Medical School, The University of Sydney, Sydney, NSW 2006, Australia

[d]Fogarty International Center, National Institutes of Health, Bethesda, MD 20892, USA

[1] To whom correspondence should be addressed: ruian@ucla.edu (RK); jlloydsmith@ucla.edu (JOLS)

1. The simplified ordinary differential equation (ODE) model for tDP transmission dynamics

To gain insights into the key factors that drive the transmission dynamics of tDP with DENV-1, we simplified the full ODE model. In the simplified model, we ignore the seasonal forcing on the mosquito population and set the rates of super-infection (infection of already infected individuals, shown as dashed lines in Fig. 2) to be 0. These assumptions give rise to the system of ODEs shown in Table S8.

Table S8. ODEs for the simplified model.

$$\frac{dS}{dt} = (N_H - S) \cdot \mu_H - \frac{\beta \cdot I_V \cdot S}{N_H} - (Q + W_V) \cdot \frac{\beta \cdot D_V \cdot S}{N_H}$$

$$\frac{dE}{dt} = \frac{\beta \cdot I_V \cdot S}{N_H} + \frac{Q \cdot \beta \cdot D_V \cdot S}{N_H} - (\sigma_H + \mu_H) \cdot E$$

$$\frac{dG}{dt} = \frac{W_V \cdot \beta \cdot D_V \cdot S}{N_H} - (\sigma_{H,D} + \mu_H) \cdot G$$

$$\frac{dI}{dt} = \sigma_H \cdot E - (\gamma_H + \mu_H) \cdot I$$

$$\frac{dD}{dt} = \sigma_{H,D} \cdot G - (\gamma_{H,D} + \mu_H) \cdot D$$

$$\frac{dR}{dt} = \gamma_H \cdot I + \gamma_{H,D} \cdot D - \mu_H \cdot R$$

$$\frac{dS_V}{dt} = (N_V - S_V) \cdot \mu_V - \frac{\beta \cdot I \cdot S_V}{N_H} - (Q + W_H) \cdot \frac{\beta \cdot D \cdot S_V}{N_H}$$

$$\frac{dE_V}{dt} = \frac{\beta \cdot I \cdot S_V}{N_H} + \frac{Q \cdot \beta \cdot D \cdot S_V}{N_H} - (\sigma_V + \mu_V) \cdot E_V$$

$$\frac{dG_V}{dt} = \frac{W_H \cdot \beta \cdot D \cdot S_V}{N_H} - (\sigma_{V,D} + \mu_V) \cdot G_V$$

$$\frac{dI_V}{dt} = \sigma_V \cdot E_V - \mu_V \cdot I_V$$

$$\frac{dD_V}{dt} = \sigma_{V,D} \cdot G_V - \mu_V \cdot D_V$$

2. Parameter estimation

Data

The frequency of dually infected individuals increased rapidly during 2001 and 2002 in Myanmar [1]. Among 9 individuals tested in 2001, 5 individuals were only infected by DENV-1, and 4 dually infected by DENV-1 and tDP; all 5 individuals tested in 2002 were dually infected (Table S9). These data, together with the month of sampling for each individual, are used to infer parameter values in the model in a maximum likelihood framework as described below. Because the number of data points is limited, we applied an additional criterion to restrict the parameter sets to those that broadly match the observed dynamics. The tDP was present at high frequency in the last individual sampled in Myanmar, in December 2002. Thus we imposed the criterion that the stop-codon lineage could not be extinct at the end of 2002. The parameter values corresponding to a given run were considered in our analysis only if the number of dually infected individuals at the end of 2002 is greater than 1. This criterion sets an upper bound for the value of $R_{eff,co}$, because simulations with high values of $R_{eff,co}$

exhibited extinction of tDP in the year 2002, due to depletion of susceptible individuals following the 2001 epidemic.

Table S9. Dates and numbers of DENV-1 infected cases in Myanmar during 2001 and 2002 according types of infection.

Data point (i)	Date of isolation	No. of individuals infected by DENV-1 only (I) -	No. of dually infected individual (D)
**1 (Mosquito)**	June, 2001	1	3
**2 (Human)**	May, 2001	1	0
**3 (Human)**	Jul, 2001	1	1
**4 (Human)**	Sep, 2001	1	0
**5 (Human)**	Oct, 2001	1	0
**6 (Human)**	Jul, 2002	0	2
**7 (Human)**	Aug, 2002	0	1
**8 (Human)**	Sep, 2002	0	1
**9 (Human)**	Dec, 2002	0	1

Model assumptions

For parameter estimation, we used the full model, with *P* and *Q* set to 0 and other parameters (except those being estimated) fixed at values used in the full model (Table S8).

To compare simulation results with data, we determined a mapping between simulation time and calendar time (Table S10), such that there was qualitative correspondence between simulation results and data reported during 1999 and 2002 when DENV-1 was the dominant serotype [2]. This mapping was determined using two criteria. First, to model endemic dengue transmission in Myanmar [2], we dropped the first 30 years of the simulation to allow dynamics to stabilize and eliminate periods when the number of dengue cases dropped below 1 during the troughs after major epidemic seasons. This is a phenomenological approach to capture the influence of population immunity on dengue dynamics. Second, we chose the first two-year period in the simulation during which the dengue incidence exhibits two consecutive peak years and mapped these onto calendar years 2001 and 2002 (Table S10), based on the historically high number of dengue cases reported in 2001 and 2002 [2]. This mapping process is *ad hoc*, but yields conservative estimates of $R_{eff,co}$ as long as the condition of endemic dengue transmission is fulfilled (see below for the sensitivity analysis for this mapping).

Table S10. The simulation times that correspond to calendar year 2001 in models with different seasonal forcing parameters.

Seasonal Forcing parameter (a)	Simulation time for year 2001 (t_{2001})
0.6	43
0.7	44
0.8	47

Maximum likelihood estimation (MLE)

The likelihood model for the data shown in Table S9 is calculated by considering the distribution of cases obtained in each month that samples were collected. Two types of cases are considered: single infections with DENV-1 and dual infections with both DENV-1 and tDPs. The negative log-likelihood function (Γ) is then:

$$\Gamma = -log_{10}(\prod_{i=1}^{9} f_i(x_{i,1}, x_{i,2}; p_{i,1}, p_{i,2}))$$

where f_i is the probability density function of the binomial distribution for the ith data point, $x_{i,1}$ and $x_{i,2}$ are the observed cases for i^{th} data point in the two categories of cases, respectively and $p_{i,1}$ and $p_{i,2}$ are the probabilities of detecting cases in the two categories shown in Table S9, respectively, for the ith data point. $p_{i,1}$ and $p_{i,2}$ are calculated from the simulation model from the proportions of individuals in the I and D compartments for humans, or the I_V and D_V compartments for mosquitoes, for a given parameter set.

The negative log-likelihood function was then minimized using the constrained parameter optimization routine fmincon in Matlab 2009b (Mathworks, Inc.). In the optimization, the parameter value t_{emg} was constrained between Mar. 1999 and Feb. 2001 according the estimated time of emergence (Fig. 1B). Other parameter values are unconstrained. Due to the nonlinearity in the likelihood function, we performed 100 runs of optimization for each parameter set using randomly generated starting values within the range of parameter variation. To confirm that the parameter values estimated to give the maximum likelihood in the 100 runs correspond to a global optimum, we performed a further 400 runs with the same settings as the first 100 runs. The maximum likelihood did not change with the additional 400 runs, suggesting the algorithm is stable and it is a global optimum. Comparison between data and simulation results using the maximum likelihood parameter values are shown in Fig. S2. The 95% confidence interval was estimated for each parameter (except t_{emg}) using likelihood profiling [3]. The estimated 95% confidence intervals are shown in brackets in Table 1, Table S3 and Table S4.

3. Sensitivity analysis

Seasonality

To assess the robustness of the MLEs to variations in the seasonal forcing parameter a in our model, we performed MLE for two other values of a: $a=0.6$ and 0.8. These two parameter values were considered because simulated dengue infection dynamics with a in the range between 0.6 and 0.8 agree with the seasonal variations observed in Myanmar [4]. The resulting MLEs are shown in Table S3 and S4. The estimated values of the parameters and their corresponding values of $R_{eff,co}$ are similar for the three choices of values of a. Therefore, the MLEs are robust to the assumptions of the seasonality parameter a.

Mapping between simulation time and calendar time

In our simulation analysis, we have chosen the first double-peak years after dengue transmission entering the endemic phase to correspond to the calendar year 2001. To assess the robustness of the MLEs to changes in this mapping scheme, we performed maximum likelihood estimation for three other mapping schemes in which later double-peak years were chosen. The dengue incidence exhibits peaks every ten years in the simulation. For our main results we mapped simulation year 44 to calendar year 2001 (t_{2001}=44); in the sensitivity analysis, the simulation years 46, 48, 54, 64 and 74 were chosen (t_{2001}=46, 48, 54, 64 or 74). These different choices correspond to different phases in the multi-year epidemiological dynamics of dengue, but the resulting dynamics of tDP are robust. In the simulations with t_{2001}=46 or 48, tDP rose to a high frequency when the prevalence of dengue is low and decreasing, whereas in the simulations with t_{2001}=54, 64 or 74, tDP rose to a high frequency

when dengue is entering an epidemic phase, similar to the epidemiological context used for our main analysis. As shown in Table S5, the estimated values of $R_{eff,co}$ are broadly similar across these choices, and fall in a narrow range of 1.23-1.36. The simulated dengue infection dynamics and the fit to the data are similar for all choices of temporal mapping (Fig. S3).

Looking closely, the estimates of $R_{eff,co}$ are slightly higher in mapping schemes where later simulation times are used. This can be understood intuitively as follows. The time required for dually infected individuals to rise from a low frequency to a high frequency is determined by the difference in the efficiency of transmission by dually infected versus singly infected individuals, i.e. $R_{eff,co}$, as well as the initial frequency of dually infected individuals among all infected individuals (Fig. S2B). Thus the estimated value of $R_{eff,co}$ must be higher if the initial frequency of dually infected individuals (among all infected individuals) is lower, given that tDP infection rises from a low frequency to a high frequency in 2-4 years (as estimated in this study, Fig. 1B). The initial frequency of dually infected individuals is driven by the prevalence of DENV-1 infections at the time when tDP first emerges. For schemes that map later simulation times to the calendar year 2001, the numbers of DENV-1 infected individuals are higher, since the ODE system gradually approaches equilibrium at later time points. Therefore the initial frequency of dually infected individuals is lower, and the estimated values of $R_{eff,co}$ are higher.

The estimated fold increases in overall DENV-1 cases are notably higher in mapping schemes where a later simulation time is used (Table S5). This is because, although introduction of the tDP leads to outbreaks of similar magnitude for the three schemes, dengue outbreaks in the absence of the tDP are smaller at later time points in the simulation (Fig. S4). Thus, the relative increases in overall DENV-1 cases are higher if we map later time points to the calendar year 2001.

Therefore, the mapping used in the main analysis yields estimates of $R_{eff,co}$ and increases in overall DENV-1 cases that are conservative, in the sense that they err on the low side in estimating the increased transmission potential of co-transmission and the increased incidence of dengue cases.

4. Probability of fixation by genetic drift

In this section, we calculate the probability that the rise of tDP frequency observed in the data could have been a result of neutral genetic drift, i.e. the scenario that W=1 and the efficiency of co-transmission of tDP and DENV-1 is identical to that of transmission of DENV-1 only, so that the rise in frequency of co-infection arose strictly by chance. Because super-infection events account for a very small fraction of tDP transmission (Fig. 3B in the main text), we ignore this transmission route in this analysis. Under this approximation, we can make an analogy to classical population genetics, and treat the dually infected individual as a separate allele in a population of infected individuals. We can use a Wright-Fisher model to calculate the expected frequency distribution of dually infected individuals over time in a population of infected individuals. Using this frequency distribution, we can calculate the probability that the observed change could have arisen through genetic drift.

The number of generations of tDP transmission

First, we estimate the generation time for transmission of dengue virus, and calculate the number of transmission generations that occurred between the emergence of tDP and our observed data. The mean latent period and mean infectious period for dengue in humans are 5 and 6 days, respectively [5]. If we assume that the time to infect the next host is uniformly distributed over the infectious

period, the mean generation time for dengue transmission is 8 days (5+6/2=8). For mosquitoes, the mean incubation period and mean lifespan are 10 and 14 days, respectively [5]. Exact calculation of the infectious period for mosquitoes is complicated, and depends on the distributions of the lifespan and incubation period (and on temperature, and other factors), but the mean will fall between 0 and 4 days, giving a mean generation time for mosquitoes of 10-12 days. Thus, averaging over humans and mosquitoes, the mean generation time for dengue transmission is approximately 9-10 days. Here, to be conservative in our estimates (in terms of calculating the maximum number of generations and thus estimating the upper bound of the probability of tDP rising to a high frequency), we use a mean generation time of 9 days in the calculation below. (We also note that our ultimate conclusion is robust to this choice of generation time.) Based on the calculated mean generation time for dengue transmission and the estimated time of tDP emergence (Feb. 2000, with 95% credible interval of Jun. 1998 to Feb. 2001, as shown in Fig. 1B in the main text), we estimate the total number of tDP transmission generations by the end of Dec. 2002 to be approximately 115 generations, with 95% credible interval of 74-182 generations.

The effective number of DENV-1 infections (N_e)

Since DENV-1 was endemic in Myanmar during the years 1998-2002, the number of DENV-1 infected individuals should be very large given the large population size in the country (around 45 million at that time). Intuitively, it is almost impossible for dually infected individuals to rise to 50% in the population within 182 generations after emergence, in a population of this size. To investigate this probability more quantitatively, we first estimate the effective number of DENV-1 infections (N_e) from sequence data using BEAST [6]. This effective number of infections can be derived from dividing the composite parameter, *popSize*, estimated in BEAST by the generation time of DENV-1 transmission. Since *popSize* is estimated based on coalescence in BEAST [6], the effective number of infections is equivalent to the effective population size in population genetic models. Therefore, it can be used directly in our Wright-Fisher model to calculate the probability for dually infected individuals to rise to a frequency of 50% in the population. Note that the effective number of DENV-1 infections may not reflect the true number of DENV-1 infected individuals. It has been shown that the relationship of these two quantities is dependent on the phase of the disease epidemic [7].

We first extracted 18 sequences derived from 18 infected individuals in Myanmar during the period from 1996 to 2001 [2] from the Genbank database (accession numbers: AY588272, AY588273, AY600860, AY606062, AY618210, AY618211, AY618877-AY618880, AY620946-AY620953). We then estimated the effective number of DENV-1 infections from 1998 to 2001 based on these 18 sequences using the Bayesian skyline coalescent model in BEAST [6] with the GTR+I+Γ_4 substitution model [8]. 3,000,000 states were collected from the MCMC chain and the first 300,000 states were excluded as burn-in. The effective sample size for each estimated parameter was checked using Tracer v1.5 to ensure convergence [9], with statistical uncertainty reflected in values of the 95% Highest Probability Density (HPD). Note that we did not use the consensus DENV-1 sequences and phylogenetic tree shown in Fig. 1 for this estimation, for two reasons. First, Fig. 1 shows consensus sequences for each lineage within each individual, i.e. an individual carrying several lineages is represented by multiple sequences in the tree. Second, in 2002, all sequences obtained came from the clade corresponding to dually infected individuals. If this high frequency of dually infected individuals is a result of higher transmission potential (as we have argued in this study), then including these sequences would violate the assumption of neutrality needed to estimate N_e by this approach. Both of these problems would bias the estimation of N_e.

To calculate N_e, we then divided the estimated values of the parameter, popSize, from BEAST by the generation time of DENV-1 transmission assumed in our study, i.e. 9 days. The estimated N_e in

Myanmar from Jun 1998 to the end of 2001 spans values in the range of 7848 to 9079 (Table S11). Although the 95% credible intervals are large, the lowest bounding value of N_e is 735 (in Jun. 1998). As we will show below, the probability of neutral drift is extremely low even if N_e is assumed to take this limiting value of 735.

Table S11. The mean and 95% credible intervals of the effective number of DENV-1 infections (N_e) of DENV-1 from 1998 to 2001.

Time	Mean (N_e)	Upper bound	Lower bound
**Dec, 2001**	9079	39411	1005
**Jun, 2001**	9075	39341	1018
**Jan, 2001**	9057	39256	1066
**Jun, 2000**	8951	38751	1099
**Jan, 2000**	8791	37904	1063
**Jun, 1999**	8556	37256	969
**Jan, 1999**	8269	36408	856
**Jun, 1998**	7848	34725	735

The probability of fixation by genetic drift

The data show that 5 out of 5 individuals sampled in 2002 were dually infected [1]. Assuming binomial sampling, the lower bound of the 95% confidence interval for the estimated frequency of dually infected individuals is roughly 50%. Here we make the most conservative assumption, and make our calculation based on this lower bound value, i.e. that the frequency of dually infected individuals reached 50% at the end of 2002. As shown above, the number of transmission generations between the emergence of tDP and the end of 2002 is approximately 115 (with 95% credible interval from 74 to 182 generations).

Now, by describing the process of DENV-1 transmission using a Wright-Fisher model, we calculate the frequency of dually infected individuals over time for different effective numbers of DENV-1 infections. Specifically, we treat the emergence of the trait of 'dually infected individual' as a newly introduced allele in the population of DENV-1 infected individuals, with an initial allele frequency of $1/N_e$. Under the Wright-Fisher model, the probability that the population size of a neutral allele at generation t+1 (X_{t+1}) is j, given that its size is i at generation t ($X_t=i$) and the effective numbers of DENV-1 infections is constant at N_e, follows the binomial distribution:

$$P(X_{t+1} = j \mid X_t = i) = B(j; N_e, i / N_e)$$

Where $B()$ denotes the binomial probability.

Therefore the probability that the population size of the allele is j at generation t+1 is:

$$P(X_{t+1} = j) = \sum_{i=1}^{N_e} B(j; N_e, i / N_e) \cdot P(X_t = i) \qquad \text{(S1)}$$

Based on Eqn. (S1), we can calculate the probability that the frequency of dual infection rises from $1/N_e$ to 50% for each generation for a given population size (N_e).

Fig. S4 shows this probability under genetic drift for a range of effective numbers of DENV-1 infections, and for three possible dates of emergence of the tDP lineage. Even when we take the most conservative limit of each quantity, i.e. the smallest estimate of N_e (735), the earliest emergence date (Jun 1998), and the lowest observed frequency (50%), we still estimate a very low probability (on the order of 10^{-4}) that the observed increase arose by neutral genetic drift. If we

assume more central values for each of these quantities, we estimate much lower probabilities ($<10^{-30}$, not shown in Fig. S4 because it corresponds to N_e values of ~8000 as shown in Table S11). This probability decreases rapidly as the number of transmission generations decreases (i.e. the date of tDP emergence becomes more recent) and as the effective number of DENV-1 infections increases. Of course the probability is also lower if we consider a rise to 100% frequency instead of 50%.

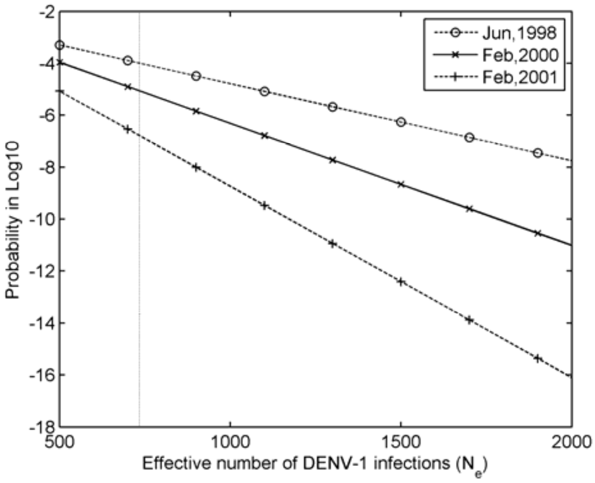

Figure S4. The probability (in Log10) that genetic drift causes the frequency of dually infected individuals to rise to 50% by the end of 2002 is extremely low given the estimated effective number of DENV-1 infections (N_e). This probability was calculated for a population that goes through 74, 115 and 182 generations, corresponding to the estimated time of tDP emergence in Feb. 2000, and the upper and lower bounds of the 95% credible interval (Feb. 2001 and Jun. 1998). The vertical gray line shows N_e=735 (the lower bound of all 95% credible intervals for the estimated value of N_e in Jun, 1998).

In summary, even with the most conservative assumptions, our calculations show that the observed rise of tDP frequency is extremely unlikely to have resulted from the stochastic process of genetic drift. This analysis supports the findings of our deterministic analysis in the main text, which shows that the rise in frequency of tDP can be explained if co-transmission of DENV-1 and tDP is considerably more efficient than transmission of wild-type DENV-1 alone.

Supporting References

1. Aaskov J, Buzacott K, Thu HM, Lowry K, Holmes EC (2006) Long-term transmission of defective RNA viruses in humans and Aedes mosquitoes. Science 311: 236-238.
2. Thu HM, Lowry K, Myint TT, Shwe TN, Han AM, et al. (2004) Myanmar dengue outbreak associated with displacement of serotypes 2, 3, and 4 by dengue 1. Emerg Infect Dis 10: 593-597.
3. Bolker BM (2008) Ecological Models and Data in R: Princeton University Press.
4. Naing CM, Lertmaharit S, Naing KS (2002) Time-Series Analysis of Dengue Fever/Dengue Haemorrhagic Fever in Myanmar since 1991. Dengue Bulletin 26: 24-32.
5. Wearing HJ, Rohani P (2006) Ecological and immunological determinants of dengue epidemics. Proc Natl Acad Sci U S A 103: 11802-11807.
6. Drummond AJ, Rambaut A (2007) BEAST: Bayesian evolutionary analysis by sampling trees. BMC Evol Biol 7: 214.
7. Frost SDW, Volz EM (2010) Viral phylodynamics and the search for an 'effective number of infections'. Philosophical Transactions of the Royal Society B-Biological Sciences 365: 1879-1890.
8. Drummond AJ, Ho SY, Phillips MJ, Rambaut A (2006) Relaxed phylogenetics and dating with confidence. PLoS Biol 4: e88.
9. Rambaut A, Drummond AJ (2007) Tracer v1.4, Available from http://beast.bio.ed.ac.uk/Tracer.
